# COVID-19 Resilience and Risk Reduction Intervention in Rural Populations of Western India: Retrospective Evaluation

**DOI:** 10.2196/47520

**Published:** 2024-07-29

**Authors:** Saurav Basu, Meghana Desai, Anup Karan, Surbhi Bhardwaj, Himanshu Negandhi, Nitin Jadhav, Amar Maske, Sanjay Zodpey

**Affiliations:** 1 Indian Institute of Public Health-Delhi Public Health Foundation of India Gurugram India; 2 Department of Monitoring, Evaluation, and Learning Bharatiya Jain Sangathana Shantilal Muttha Foundation Pune India

**Keywords:** COVID-19, community intervention, COVID-19 vaccination, COVID-appropriate behavior, impact evaluation, rural health, community-based intervention, rural population, awareness, disease prevention, health literacy, public health, pandemic, digital health, community awareness, effectiveness, low- and middle-income countries, vaccination, infodemic

## Abstract

**Background:**

Globally, especially in the low- and middle-income countries (LMICs), rural populations were more susceptible to the negative impact of the COVID-19 pandemic due to lower levels of community awareness, poor hygiene, and health literacy accompanying pre-existing weak public health systems. Consequently, various community-based interventions were engineered in rural regions worldwide to mitigate the COVID-19 pandemic by empowering people to mount both individual and collective public health responses against the pandemic. However, to date, there is paucity of information on the effectiveness of any large-scale community intervention in controlling and mitigating the effects of COVID-19, especially from the perspective of LMICs.

**Objective:**

This retrospective impact evaluation study was conducted to evaluate the effect of a large-scale rural community–based intervention, the COVID-Free Village Program (CFVP), on COVID-19 resilience and control in rural populations in Maharashtra, India.

**Methods:**

The intervention site was the rural areas of the Pune district where CFVP was implemented from August 2021 to February 2022, while the adjoining district, Satara, represented the control district where the COVID-Free Village Scheme was implemented. Data were collected during April-May 2022 from 3500 sample households in villages across intervention and comparison arms by using the 2-stage stratified random sampling through face-to-face interviews followed by developing a matched sample using propensity score matching methods.

**Results:**

The participants in Pune had a significantly higher combined COVID-19 awareness index by 0.43 (95% CI 0.29-0.58) points than those in Satara. Furthermore, the adherence to COVID-appropriate behaviors, including handwashing, was 23% (95% CI 3%-45%) and masking was 17% (0%-38%) higher in Pune compared to those in Satara. The probability of perception of COVID as a serious illness in patients with heart disease was 22% (95% CI 1.036-1.439) higher in Pune compared to that in Satara. The awareness index of COVID-19 variants and preventive measures were also higher in Pune by 0.88 (95% CI 0.674-1.089) points. In the subgroup analysis, when the highest household educational level was restricted to middle school, the awareness about the COVID-control program was 0.69 (95% CI 0.36-1.021) points higher in Pune, while the awareness index of COVID-19 variants and preventive measures was higher by 0.45 (95% CI 0.236-0.671) points. We did not observe any significant changes in the overall COVID-19 vaccination coverage due to CFVP implementation. Furthermore, the number of COVID-19 deaths in both the sampled populations were very low. The probability of observing COVID-19–related stigma or discrimination in Pune was 68% (95% CI 0.133-0.191) lower than that in Satara.

**Conclusions:**

CFVP contributed to improved awareness and sustainability of COVID-appropriate behaviors in a large population although there was no evidence of higher COVID-19 vaccination coverage or reduction in mortality, signifying potential applicability in future pandemic preparedness, especially in resource-constrained settings.

## Introduction

The global health crisis caused by the COVID-19 pandemic affected communities worldwide, with poverty and social inequity accentuating vulnerability to risk of infection and death, particularly in low- and middle-income countries (LMICs) [1]. In low-income nations, rural populations were more susceptible to the negative impact of COVID-19 due to lower levels of community awareness, poor hygiene, and lower health literacy accompanying pre-existing weak public health systems [2,3]. A multi-country analysis from the Southeast Asian region reported high prevalence of suboptimal COVID-appropriate practices [4]. Furthermore, the challenge of health care accessibility in these settings was exacerbated, as a significant proportion of health facilities lacked routine service provision functionality either due to infrastructural deficits or due to the diversion of existing health resources for the COVID-19 pandemic management purposes [5]. In India, similar challenges were experienced in the rural countryside, which constitutes nearly 70% of the country’s population [6]. A study from the South Indian state of Karnataka reported high levels of stressors among village communities during the COVID-19 pandemic due to financial distress and perceived inadequacy of specific treatment availability for COVID-19 [7,8]. COVID-19 vaccination, which was the mainstay for preventing severe COVID-19 and reduction of mortality, was frequently lagging in rural areas compared with their urban counterparts due to factors such as reduced awareness, vaccine hesitancy, and difficulty in access to vaccination services [9]. Rural health systems, particularly in LMICs, were not equipped to handle the COVID-19 pandemic on a global scale. Additionally, national responses to COVID-19 generally failed to adequately address the unique challenges faced by rural communities [10].

In the midst of the escalating COVID-19 spread, various community-based interventions were engineered in rural areas across several LMICs to restrict the transmission of COVID-19 and enable and empower the people to mount an effective response against disease transmission by adopting standard preventive measures [11-13]. These intervention methods were predominantly driven by the principles of community engagement through dedicated village level volunteers who coordinated government and administrative action with the perceived village requirements to enable need-based assistance to the village communities. Technical support was also significantly leveraged especially through digital communication via SMS text messages and prerecorded voice calls to improve COVID-19–related awareness in rural communities [14,15]. The effective application of social media platforms to improve awareness and behaviors that were protective against COVID-19 were also reported by studies in Jordan and Bangladesh [15,16].

There is emerging evidence indicative of the nature of successful COVID-19 initiatives in rural areas in collaboration with local communities, sharing encouragement for multisectoral outlays, with engagement of local cultures and their leaders [10]. However, till date, there is paucity of information on the effectiveness of any large-scale community intervention in controlling and mitigating the effects of the COVID-19 pandemic, especially from the perspective of LMICs. Our study was therefore conducted to assess the impact of a large-scale community intervention for promoting COVID-19 resilience in rural areas of the Pune district in India.

The state of Maharashtra in India was severely affected by the COVID-19 pandemic, especially in the aftermath of the peak of the second (Delta) wave of the COVID-19 pandemic, in response to which, the state government launched a COVID-Free Village Scheme (CFVS) cum contest in June 2021 for the empowerment of villagers to combat the COVID-19 pandemic, and this scheme is one of the largest programs in the world [17]. The CFVS envisaged collective action for pandemic control through the creation of multiple village level groups known as village task force (VTF) within each gram panchayat, which represents the local grassroots–governing institute at the level of villages in India. Each constituted VTF was entrusted with specific responsibilities toward mitigation and resilience to COVID-19 infection in all villages. Select nongovernmental organizations (NGOs) in few districts were also entrusted with the responsibility of planning and implementing CFVS in certain districts by providing technical and managerial support.

The Pune district in Maharashtra reported nearly 1.1 million confirmed COVID-19 cases, with over 19,000 deaths until March 2022. Rural populations in the district were particularly vulnerable, since Pune, the seventh largest city in India, recorded a high burden of COVID-19 infection during the initial waves of the pandemic [18]. The CFVS was implemented in the rural areas of Pune district from August 2021 to March 2022 through support with a grassroots NGO (Bharatiya Jain Sangathana) that provided technical support and coordination with the district and village administration to fulfil community needs and achieve community mobilization during the pandemic.

Assessment of the CFVS in Pune district would explain the usefulness of such large community-based interventions in controlling the COVID-19 pandemic and suggest the effect and applicability of the model in combating future pandemics and other endemic public health problems. Inclusive of such additional components, the scheme in Pune with the operational nomenclature of the COVID-Free Village Program (CFVP) was differentiable from the CFVS implemented in other districts of the state. This impact evaluation was therefore conducted to understand the difference in COVID-19 awareness, adherence to protective measures, vaccination coverage, morbidity and mortality, and associated factors between the CFVP (intervention) and the CFVS (comparison) villages.

## Methods

### Study Settings

The intervention sites were the rural areas of the Pune district where CFVP was implemented from August 2021 to February 2022, while the adjoining district, Satara, represented the control district where CFVS was implemented. Satara was among the 5 districts of Maharashtra that had the highest COVID-positive case rate, and the reported caseload in Satara was higher than the Maharashtra state average. As per the 2017-2018 report of the Public Health Department of Maharashtra, there were 66 primary health centers and 353 subcenters for 81.01% (2,433,363/3,003,741) of the rural population in the Satara district. In comparison, the Pune district was the worst affected during the pandemic and recorded the highest number of COVID-19 cases in Maharashtra. There were significant differences in the availability and accessibility of health facilities between the urban and rural areas in these districts. For 39.01% (3,678,226/9,429,408) of the rural population in Pune district, only 86 primary health centers and 358 subcenters were available across the district. Pune zilla parishad (elected district councils in rural India) collaborated with various NGOs and community organizations to implement COVID-19 control measures, including setting up vaccination centers, conducting testing drives, and providing health care.

The government of Maharashtra, in its advisory, recommended the launch of the CFVS requiring the constitution of 5 VTFs in every village for the conduct of household surveys (VTF-1), functioning of isolation centers (VTF-2), arranging vehicle drivers for transporting patients for COVID testing and treatment (VTF-3), running a COVID helpline (VTF-4), and promoting COVID-19 vaccination (VTF-5). The CFVS in Pune was supported by an NGO (Bharatiya Jain Sangathana) that provided additional COVID-19 containment and resilience components toward the planning, implementation, and community mobilization, especially the youth; thus, this scheme was operationally defined as CFVP to distinguish it from the CFVS that was implemented in Satara sans NGO involvement. Furthermore, the VTFs created as part of the CFVP differed from the CFVS by incorporating support for COVID awareness and behavior change in the rural population. CFVP VTFs comprised VTF-1 that focused on COVID-appropriate behaviors (masking, handwashing, social distancing); VTF-2 on tracing, tracking, testing, and treatment; VTF-3 that was dedicated toward establishing COVID care centers and quarantine centers; VTF-4 on providing awareness and support to enroll for government schemes; and VTF-5 that was dedicated to work on COVID-19 vaccination. There was flexibility in the CFVP approach, as the task forces were designed to be activated and responsive to ground level exigencies due to the dynamic COVID situation. The CFVP functions in Pune consisted of (1) a hybrid approach for deployment of traditional information, education, and communication (IEC) platforms in conjunction with social media, especially instant messaging through WhatsApp; (2) planning and implementation of camp-based approaches for COVID-19 testing and vaccination as per perceived needs of the community through support from dedicated Taluka (subdistrict) coordinators stationed by the NGO; and (3) deploying access to a multilingual knowledge management system (KMS) platform for web-based training of village level stakeholders, especially VTF members, and having a repository of pandemic management information resources issued by both government and nongovernmental public health organizations (Multimedia Appendix 1). The CFV-KMS portal served as a one-stop-shop for program stakeholders to easily access and download user-friendly program resources in their preferred language at any time. During the pandemic when the movement of people was restricted, KMS provided instant access to program resources for self-learning, awareness generation, and capacity building. Detailed processes, roles and responsibilities of stakeholders, audiovisual learning modules, pictorial guidebooks, and behavior change communication/IEC material on the portal helped people to better understand the project and know what was expected of them. Program resources available on the portal were also extensively used by master trainers to conduct daily web-based training of sarpanches, VTF members, and other stakeholders.

A logic model guided by a theory of change in the CFVP envisaged an increase in the adaptive, absorptive, and transformative capacity of the communities mediated by principles of civic mindedness and social responsibility toward strengthening individual agency for adopting positive COVID-appropriate behaviors and instilling resilience (Multimedia Appendix 2). In this context, the vulnerable community on having improved accessibility to resources was expected to develop increased COVID-19 awareness, which enables adherence to COVID-appropriate behaviors translating into increased COVID-19 vaccination acceptability and confidence, reducing viral transmission, and promoting positive health-seeking behaviors for reducing COVID-related hospitalizations and deaths.

### Study Design

This analysis reports the quantitative results of a large observational mixed methods concurrent triangulation study conducted from May 2022 to July 2022. In this retrospective impact evaluation, quantitative data were collected from sample households from villages across intervention (CFVP) and comparison (CFVS) arms. The CFVS implemented the recommendations of the government for maintaining COVID-free villages without any external support, while the CFVP implemented the same with support from the NGO, as described above. We created a matched sample of nonexposed (to CFVP) villages and households from an adjoining district, Satara, a predominantly rural district sharing its north border with Pune. Satara has a total population of 3,003,741, with 81.01% (2,433,031/3,003,741) being rural. Pune has a total population of 9,431,349, with 38.9% (3,678,226/9,431,349) being rural [19,20].

### Sampling

Sample size estimations were based at 5% significance level (α error), 90% power, expecting 85% success in intervention (CFVP) and 80% success in comparison group (CFVS), with 20% nonresponse rate. A minimum of 1500 households in the intervention and control district each were required for estimating the change in outcomes. Additional 500 households were selected in the control district to account for any loss of power after the matching analysis. For the household survey, a random 2-stage stratified sampling was followed. In the first stage, all villages in Pune (n=1173) and Satara (n=1668) districts were separately stratified into 4 equal strata based on the average number of households per village, with stratum 1 containing the smallest number of households per village and stratum 4 containing the largest number of households per village. A total sample of 1500 households in the Pune district were allocated to each group, with a varied proportion (50% households per village from stratum 1, 40% from stratum 2, 30% from stratum 3, and 20% for stratum 4), which determined the minimum number of villages to be covered in each stratum and in Pune. Equal number of villages, with similar socioeconomic and village infrastructure status, were selected from each stratum in Satara. Similarity in the socioeconomic and infrastructure status of villages in each stratum was determined through a statistical matching exercise. A total of 2000 households were allocated to the selected villages in Satara (control) district in the same proportion as in the Pune district. In the second stage, households from each village were selected using a systemic random sampling method. A household list in each village was obtained with the help of local authority, and households were systematically selected from the list by using varied proportions (50% households per village from stratum 1, 40% from stratum 2, 30% from stratum 3, and 20% for stratum 4) in villages. We also provided scope of replacement of households in case of nonresponse in the control villages.

### Recruitment

Data collection was conducted from the month of May to June 2022 by an external survey agency that had no role in the implementation of the CFVS or CFVP. A total of 25 field investigators along with 5 supervisors and 2 qualitative researchers were involved in the process of data collection. Field investigators were provided 3 days residential training before the commencement of the data collection where they were explained the purpose of the CFV project, household selection method, consent procedures, and administration of the tool. After 2 days of classroom training, a 1-day field visit was planned for the participants to an environmental-sensitive area to familiarize the trainees on how to implement the tool in real-world situations. Quality assurance in data collection was assessed by the first and third authors through independent supervisory field visits and virtual meetings and feedback from the data enumerator teams. Data were collected using a close-ended interview schedule prepared in English that was translated into Marathi and Hindi languages and that had distinct thematic sections for capturing information associated with each VTF function (Multimedia Appendix 3).

Interviews were conducted with an adult member of the household who was preferably the head of the household or the individual having the highest educational qualification among all household members, although due to operational challenges, the adult household member available and willing to participate was also interviewed. The interview schedule collected information on the sociodemographic characteristics of the household, COVID-19 vaccination status in terms of the number of doses received, associated information such as site of initial vaccination, distance from the vaccination site, awareness regarding severity of COVID-19 infection, awareness and adherence to COVID-appropriate behaviors, utilization of the Aarogya Setu official government contact tracing and awareness app, accessibility and utilization of COVID-19 testing services, COVID-19 morbidity related to hospitalization, COVID-related government scheme utilization, etc. The interview schedule was pretested in 50 households in a different intervention village to assess participant comprehension, and further changes were made in few items based on the analysis of the responses and feedback from the field investigators.

### Study Measures and Analysis

Two-stage matching analysis was performed to create the control groups (nonintervention population). First, the selected 16 villages in Pune (intervention/CFVP) were matched with villages in Satara (comparison/CFVS) by using a range of sociodemographic and village infrastructure indicators. These indicators were collected from the village directory of the census of India 2011. Second, within the selected villages of Pune and Satara, the households were matched using sociodemographic and health indicators, and only the matched households were further used for the impact assessment analysis.

#### Matching of Villages

Initially, we had 1745 villages for Satara district, and after removing the villages with extreme population numbers (less than 100 and more than 15,000 persons), 1668 villages were used for the matching analysis. Considering a combined list of the 16 sample villages in Pune and all the villages (after removing extremely small/large villages) in Satara, we generated the propensity score by using a logit model (Table S1 in Multimedia Appendix 4). The estimation with the logit model and the predicted propensity score used equation 1.

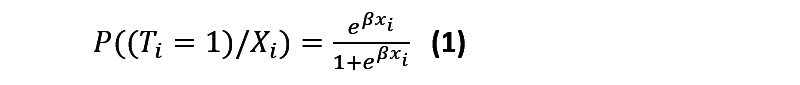



where *T_i_* indicates whether village *i* belong to Pune (intervention) district. The vector *Xi* indicates household demographic, sociodemographic, and infrastructure indicators, and β is a vector of the parameters to be estimated. Using the estimated parameters, we generated a linear prediction of the propensity score for each village. Using the nearest neighbor matching method, we selected the control villages for each intervention village. We selected 2 control villages with the nearest propensity score for each intervention village, in case replacement of village was required for any logistic reason.

#### Household Matching

Even after village-level matching of sociodemographic and infrastructural indicators, we expected some household level differences to prevail in villages. We further controlled such differences by using a household level matching in selected sample villages by using a similar exercise as mentioned in the village level matching. Matching reduced the number of sample households by about 10%, both in Pune and Satara, with 119 households in Pune and 280 households in Satara. However, there was a significant reduction in the overall imbalance of the sample (Table S2 in Multimedia Appendix 4). Logit results in household matching reflected that households in Pune were more likely to have female respondents, to be Hindu, and have a joint family and less likely to have adults older than 60 years and a family member aged 6-18 years. However, households in Pune were more likely to have at least one member with education level of primary school or above (Tables S3 and S4 in Multimedia Appendix 4).

#### Outcome Indicators

The CFVP was expected to enhance awareness in the population about COVID-19–appropriate behavior for protection against the disease. Using 13 dichotomous indicators of awareness about the COVID-19 challenges and precautions, we conducted principal component analysis to generate an awareness index of population. A similar index was also generated for awareness about the CFVP. We also generated a combined awareness index combining the 2 dimensions of awareness. This change in awareness is expected to translate into higher COVID-19 vaccination coverage and reduced mortality due to the disease.

The secondary outcome indicators for assessing the effect of the program included (1) number of deaths detected per 100,000 population; (2) COVID-19 vaccination coverage; (3) perceived effect of COVID-19 on persons with comorbidities; (4) use of preventive measures such as frequency of handwashing or sanitizing and adherence to the habit of wearing masks; (5) difficulty in accessibility and affordability of essential hygiene products; (6) use of the Aarogya Setu digital app for contact tracing, self-assessment, and understanding the risk of infection status and for knowing updates, advisory, and best practices related to COVID-19; (7) use of the vaccination facilities in public and private settings; (8) availability of transportation for the villagers when site of vaccination was farther than 1 km; and (9) regular availability of some other medical services such as regular under-3-years immunization, regular antenatal care service, access to ambulance/emergency transport for pregnant women, and access to medicines for patients with chronic diseases.

#### Estimating the Effects of CFVP

The effects of CFVP on different outcomes were estimated using matched household data (n=1381 in Pune and n=1780 in Satara) across the intervention and comparison arms. Linear regression (equation 2) and limited dependent variable (equation 3) models were used for the relevant outcomes.


y_ij_ = α + β_1_P + β_2_X_ij_ + e_ij_
**(2)**

logit (yij) = α + β_1_P + β_2_X_ij_ + є_ij_
**(3)**

where y_ij_ is the outcome of interest for households *i* living in village *j*, *P* is a dummy variable for households living in Pune, *X_ij_* are the socioeconomic and health indicators of households and *e_ij_* and є_ij_ are the usual error terms in the respective equations. β_1_ is the main parameter representing the effect of the program intervention on outcomes. We estimated β_1_ for the outcomes related to awareness index coefficient by using equation 1 and odds ratios of other outcome indicators by using equation 2 for all the other outcomes. For a robustness check of any village-specific effects, we also used village dummies as control, and we did not find any major difference in the results, except a couple of outcomes at the margin of statistical significance turning out to be statistically insignificant.

#### Subgroup Analysis

With the expectation that the effects of CFVP should be equitable and therefore higher among low socioeconomic status and vulnerable populations, we conducted 2 subgroup analyses: the first was for the socially disadvantaged subgroup comprising the scheduled caste and scheduled tribe households that were grouped and analyzed collectively, while the second subgroup comprised the members of households with maximum education up to middle school. The scheduled caste and scheduled tribe people are officially considered as representing the most socioeconomically disadvantaged groups in India [21].

### Ethics Approval

This study is compliant with the Declaration of Helsinki. Written and informed consent were obtained from all the study participants. This study was approved by the institutional ethics committee of the Indian Institute of Public Health, Delhi. Participation was voluntary, and the participants could omit answering any questions. All personal identifiers were removed prior to data analysis through the development of an anonymized data set to ensure privacy and confidentiality of the study participants. No monetary incentive was provided to any of the participants for participation in this study.

## Results

The CFVP in the rural areas of Pune district of Maharashtra conducted nearly 8 months since its inception had near universal uptake by the villages (n=1300). The sociodemographic characteristics of the participants in Pune and Satara are reported in Table S5 in Multimedia Appendix 4.

### Awareness About COVID-19 Challenges and COVID-19 Control Programs

Table 1 reports the effects of CFVP on the awareness levels of the population regarding COVID-19 variants and preventive measures and COVID-19 control programs (CFVP or otherwise). The awareness index of COVID-19 variants and preventive measures were higher in Pune (intervention) by 0.55 (95% CI 0.455-0.636) points compared with that in Satara (control). Awareness was higher in Pune for almost all indicators, with awareness about handwashing and vaccination reflecting the highest change (Table S6 in Multimedia Appendix 4). Similarly, the awareness of the COVID-control program (CFVP or CFVS) in Pune was higher by 0.22 (95% CI 0.79-0.36) points index level as compared to that in Satara. Among the different individual indicators of COVID-19 control programs (including CFVP), awareness about government initiatives such as focus awareness campaigns and awareness of public announcements and social media as methods of spreading awareness were also higher in Pune. The combined awareness level was higher in Pune by 0.43 (95% CI 0.29-0.58) points (Table 1).

**Table 1:** Retrospective impact evaluation of the effect of the COVID-Free Village Program intervention on the awareness levels of participants in the intervention district (Pune) in 2022 (N=3183).

Variable	Index for COVID-19 awareness	Index for CFVP^a^-related awareness	Index for combined awareness
Intervention district coefficient	0.546 (0.455-0.636)	0.218 (0.079-0.358)	0.430 (0.285-0.575)
*P* value	<.001	.002	<.001
Control	Reference^b^	Reference	Reference
*R* ^2^	0.1419	0.1075	0.1357

^a^CFVP: COVID-Free Village Program.

^b^Equal number of villages, with similar socioeconomic and village infrastructure status, were selected from each stratum in Satara. Similarity in the socioeconomic and infrastructure status of villages in each stratum was determined through a statistical matching exercise.

The CFVP had a positive impact on COVID-19 safety protocol in general. The probabilities of responding yes for handwashing with soap/sanitizer at least 4 times a day and wearing a mask while leaving the house were approximately 23% (95% CI 3%-45%) and 17% (95% CI 0%-38%), respectively, higher in Pune. Further, participants in Pune had significantly lower probability (30%-40% lower probability) of facing any difficulty accessing these prevention materials (Table 2).

**Table 2:** Retrospective impact evaluation of the effect of the COVID-Free Village Program on the use of preventive measures and any difficulty faced in accessing preventive materials in the intervention district (Pune) in 2022 (N=3183).

Indicators	Odds ratio (95% CI)	*P* value	Pseudo *R*^2^
Wash your hands with soap/sanitizer at least 4 times a day	1.225 (1.034-1.451)	.02	0.047
Wear the mask while leaving the house	1.169 (0.993-1.375)	.06	0.0352
Faced any difficulty in accessing or affording soap	0.688 (0.515-0.919)	.01	0.060
Faced any difficulty in accessing or affording sanitizer	0.606 (0.473-0.776)	<.001	0.056
Faced any difficulty in accessing or affording mask	0.580 (0.445-0.755)	<.001	0.057

### Perception About Effect of COVID-19 on Patients With Comorbidities

The probability of perception of COVID as a serious illness in patients with heart disease was 22% (95% CI 1.036-1.439) higher in Pune compared to that in Satara, 2.5 (95% CI 2.076-2.914) times higher in patients with hypertension, almost 2 (95% CI 1.606-2.298) times higher in patients with lung disease, and 57% (95% CI 1.339-1.854) higher in people with low immunity (Table 3).

**Table 3:** Retrospective impact evaluation of the effect of the COVID-Free Village Program on the perception of COVID-19 among patients with comorbidities in the intervention district (Pune), 2022 (N=3183).

Indicators	Odds ratio (95% CI)	*P* value	Pseudo *R*^2^
COVID-19 is more serious in people with heart disease	1.221 (1.036-1.439)	.02	0.024
COVID-19 is more serious in people with diabetes	1.059 (0.901-1.203)	.48	0.021
COVID-19 is more serious in people with hypertension	2.460 (2.076-2.914)	<.001	0.062
COVID-19 is more serious in people with lung disease	1.921(1.606-2.298)	<.001	0.049
COVID-19 is more serious in people with low immunity	1.576 (1.339-1.854)	<.001	0.060

### COVID-19 Testing

The probability of people getting tested for COVID-19 was higher in Pune by 32% (95% CI 1.126-1.553), although the odds of availability of the testing camps in the village was 84% (95% CI 0.133-0.191) lower in Pune (Table S7 in Multimedia Appendix 4).

### COVID-19 Mortality

On comparing the overall district level population estimates, Pune (rural areas) had significantly lower COVID-19 deaths per 100,000 population compared to Satara (rural areas) during the period of observation. The number of COVID-19 deaths per 100,000 population in Pune rural district in September 2021, October 2021, November 2021, December 2021, January 2022, and February 2022 was 2.5, 2.42, 1.09, 0.46, 0.68, and 0.54, respectively. In Satara, the corresponding number of COVID-19 deaths per 100,000 population in September 2021, October 2021, November 2021, December 2021, January 2022, and February 2022 was 37.11, 29.96, 1.85, 14.26, 1.81, and 0.86, respectively. Within the sample in Pune, 5 households reported death of a member in their family and 54 households reported hospitalization of a family member due to serious COVID-19 illness, while in Satara, 12 households reported death of a member in their family and 87 households reported hospitalization of a family member due to serious COVID-19 illness during the pandemic.

### COVID-19 Vaccination Coverage

On comparing COVID-19 vaccination coverage in the district level in February 2022, rural Pune had first dose coverage of 107.3% and second dose coverage of 94.7% compared to Satara with 83.1% and 71.3% coverage, respectively. On comparing COVID-19 vaccine coverage in the sample, in Pune, 79.3% (1298/1637) received 2 doses, 10.6% (173/1637) received a single dose, and 2.8% (49/1637) received a precaution (booster) dose, while in Satara, 88.4% (1806/2043) received 2 doses, 7.4% (152/2043) received a single dose, and 1.7% (35/2043) received a precaution (booster) dose. Table 4 reports the difference in the utilization of vaccination facilities across Pune and Satara. The probabilities of populations aged 60 years and older receiving vaccination at government, private, or camp facilities in Pune were lower those in Satara, but the probabilities of populations younger than 60 years receiving vaccination in all these 3 types of facilities in Pune were higher than those in Satara. The probability of the villagers being provided with transportation services if the site of vaccination was over 1 km was almost 41% (95% CI 0.976-2.035) higher in Pune, but this difference was not statistically significant (Table 4).

**Table 4:** Retrospective impact evaluation of the difference in the utilization of vaccination facilities in the intervention district (Pune) in 2022.

Indicators	Odds ratio (95% CI)	*P* value	Population, n	Pseudo *R*^2^
The first dose of COVID-19 vaccine for eligible household members aged 60 years and older was administered at the government facility	0.28 (0.239-0.335)	<.001	3183	0.0831
The first dose of COVID-19 vaccine for eligible household members aged 60 years and older was administered at the village vaccination camp	0.754 (0.622-0.893)	.001	3183	0.099
The first dose of COVID-19 vaccine for eligible household members younger than 60 years was administered at a private facility	0.315 (0.161-1.609)	.17	2128	0.168
The first dose of COVID-19 vaccine for eligible household members younger than 60 years was administered at the government facility	0.327 (0.276-0.387)	<.001	3183	0.0609
The first dose of COVID-19 vaccine for eligible household members younger than 60 years was administered at a private facility	4.826 (2.220-10.492)	<.001	3079	0.1026
The first dose of COVID-19 vaccine for eligible household members younger than 60 years was administered at the village vaccination camp	2.491 (2.112-2.940)	<.001	3183	0.0546
Transportation was provided for the villagers if the site of vaccination was farther than 1 km	1.409 (0.976-2.035)	.07	3128	0.0566

### Other Indicators

Although the installation of the Aarogya Setu contact tracing app was not statistically significant across Pune and Satara, the probability of using the app for obtaining updates, advisory, and best practices related to COVID-19 was higher in Pune by 53% compared to that in Satara. The probability of the availability of routine medical services, continuing routine child immunization, routine antenatal services, and medicines for chronic diseases had significantly higher odds ratios in Pune than those in Satara. Furthermore, the probability of observing COVID-19–related stigma or discrimination in their locality in Pune was 68% (95% CI 0.133-0.191) lower than that in Satara (Table S5 in Multimedia Appendix 4).

### Subgroup Analysis

#### Group With the Highest Household Education Level Up to Middle School

The effect of CFVP on the awareness index of COVID-19 variants and preventive measures was higher in Pune by 0.88 (95% CI 0.674-1.089) points. When the highest household educational level was restricted to middle school, the awareness about the COVID-control program (CFVS/CFVP) was also 0.69 (95% CI 0.36-1.021) points higher, with the combined awareness level of both higher by 0.95 (95% CI 0.612-1.306) points (Table S7 in Multimedia Appendix 4). The effect of CFVP on the adherence to the safety protocol such as handwashing and wearing mask was not significant in this subgroup with the highest education level up to middle school although the probability was 60% lower in experiencing difficulty in accessing soap (95% CI 38%-81%) or sanitizers (95% CI 32%-75%). The perception about COVID-19 affecting persons with comorbidities more seriously was found higher in this subgroup. The probability of the perceived seriousness of COVID was almost 6 (95% CI 3.726-9.390) times higher for patients with hypertension and 2.7 (95% CI 1.709-4.283) times higher for patients with lung disease. There was no significant difference seen in the use of Aarogya Setu app in the group except that the probability of the usage of app for knowing updates, advisory, and best practices related to COVID-19 was 2.8 (95% CI 1.02-8.042) times higher. For members aged 60 years and older, the probability of receiving vaccination in the government facility was significantly lower. The probability of eligible members younger than 60 years receiving vaccination at village camps was twice (95% CI 1.627-3.476) higher. There was a positive effect of CFVP on accessibility to other medical services. For instance, the probability of regular under-3-years immunization and regular antenatal care checkup was almost twice as high in this subgroup. Further, the probability of availability of emergency medical transport for pregnant mothers was 16% (95% CI 1.119-2.41) higher and availability of medicines to the patients with chronic diseases was 2.6 (95% CI 1.791-3.934) times higher in this subgroup (Table S8 in Multimedia Appendix 4).

#### Group With Socially Disadvantaged Households

In the group comprising socially disadvantaged households, the results were in favor of Pune for all the 3 awareness indices, though it was only significant for the awareness index of COVID-19 variants and preventive measures, which was higher by 0.45 (95% CI 0.236-0.671) points. The CFVP had a positive impact on the adherence to the safety protocol in this subgroup. The probability of washing hands with soap/sanitizer at least 4 times a day was 54% (95% CI 1.008-2.366) higher and using mask was 95% (95% CI 1.29.7-2.932) higher in this group. The perception about COVID-19 affecting persons with comorbidities more seriously was found higher in this group. The probability of the perceived seriousness of COVID in patients with lung disease was 86% (95% CI 0.756-1.762) higher and that in hypertensives was almost thrice (95% CI 1.953-4.484) higher in this group. There was no significant difference seen in the use of Aarogya Setu app. The probabilities of the eligible members receiving vaccination at government facilities were lower, but the probability of receiving COVID-19 vaccine for the eligible household members younger than 60 years at the village vaccination camp was 10 (95% CI 6.2-16.195) times higher. The probability of being provided with the transportation for the villagers if the site of vaccination was farther than 1 km was higher by 3.6 (95% CI 1.028-13.206) times. The CFVP also positively affected the availability of the other medical services. For instance, the probability of receiving regular antenatal care checkup and availability of medicines to patients with chronic diseases was 3 times higher (Table S8 in Multimedia Appendix 4).

## Discussion

### Principal Findings

Rural populations in LMICs, including India, experienced major barriers in their pandemic preparedness due to pre-existing lacunae in public health preparedness and infrastructural deficit. CFVP applied the principles of village empowerment, volunteerism, and efficient government-citizen partnership interlaced with effective supervision and management with a vibrant grassroots level partner to target community mobilization and behavior change and augment government and health system efforts. A comparison of the mortality trends in the districts indicate that Pune (rural areas) had significantly lesser COVID-19 deaths per 100,000 population compared to Satara, but this trend predated the implementation of CFVP, which negates a temporal relationship. Furthermore, the number of COVID-19 deaths in both the sampled populations were very low. Overall, district vaccination coverage in Pune (rural areas) was higher than that in Satara, but within our sample, Satara had overall slightly higher 2-dose vaccination coverage.

Our analysis suggests that rural households in Pune (intervention district) had substantially improved awareness regarding multiple aspects of COVID-appropriate behavior, higher adherence to the recommended practices such as handwashing with soap and water, and higher persistence than those in Satara that constituted the comparison group households. This finding is also confirmed by reporting of significantly increased utilization of IEC through both traditional and social media in Pune compared to the comparison site. Effective health information campaigns play a pivotal role in improving public awareness and protective healthy behaviors [22]. Previously, studies conducted during the early phase of the COVID-19 pandemic in South Asia have reported some evidence of audio messages and SMS text messages to be effective in improving the awareness and practices of rural communities, especially among women [13,22,23].

A study from rural Bihar, a low-income eastern state of India, reported that households experiencing economic problems reported higher recall of public health messages related to safe sanitation and hygiene and therefore were associated with improved COVID-appropriate behaviors related to social distancing and water, sanitation, and hygiene during the early phase of the COVID-19 pandemic [24]. A study in China also found social media to be an effective tool in promoting COVID-appropriate behaviors in the general population [25]. Our findings are also indicative of the potential of such holistic community-based interventions in the attainment of behavior change–driven public health goals during public health emergencies. Furthermore, our findings on the perceived lowering of COVID-19–related stigma in the intervention site is significant because stigmatization during the pandemic was globally known to worsen mental health, intention to vaccinate, hinder treatment access, and lower the quality of life in vulnerable populations resulting from discriminatory behaviors [25-27]. The lowering of COVID-19–related stigma in the general population therefore could have indirectly influenced several potential beneficial public health outcomes.

Although the improvement in COVID-19 awareness in Pune villages could be attributed to CFVP, a causal relationship cannot be conclusively established since the baseline knowledge, attitude, and practice information in Satara and Pune was unavailable, accentuating the risk of endogeneity [28]. Furthermore, a potential confounder was that the Pune district has higher (nearly 60%) urban demographics compared to Satara, which is mostly rural (~80%); therefore, the potential interaction of the participants from the Pune rural site with urban areas rendered them an advantage of exposure to other urban government/nongovernment-initiated COVID-19 IEC campaigns. Nevertheless, we tried to minimize this risk by matching the 2 districts based on a large number of sociodemographic indicators at the village and household level. Furthermore, sustainability of practices promotive of COVID-appropriate behavior is an important long-term indicator of community protectiveness and resilience against other infectious diseases [29,30].

Previous studies from LMICs, including India, have indicated that limited accessibility to COVID-19 vaccination services could reduce the total vaccination coverage, especially in rural and underserved communities [31,32]. Although we could not estimate the overall impact of CFVP on COVID-19 vaccination coverage in Pune, we observed some factors indicative of increased vaccination service accessibility and reduced vaccine hesitancy. For instance, in the Pune (rural areas) district sample, a significantly higher proportion of households had COVID-19 vaccination beneficiaries that were vaccinated for their first dose at the village vaccination camp, thereby reaching the unreached vulnerable populations, especially the older adults and persons with comorbidity. Furthermore, vulnerable groups, socially and educationally, benefited from the CFVP intervention at rates that were comparable to those of the socioeconomically advantaged groups, suggesting the high equitability of CFVP.

Our assessment of CFVP suggests the potential applicability of a similar model in rural community empowerment processes. Platforms such as the open-access multilingual web-based KMS knowledge portal, one of the core strategies of CFVP to address the access, awareness, and information deficiency barriers during the pandemic, can be developed for ease of access to accurate and validated health information to avoid infodemics and scaling up behavior change communication initiatives [33]. Our findings suggest that comprehensive web-based program resources can also be extensively used for the training of grassroots stakeholders in rural areas, as demonstrated within CFVP. The evidence from this study is also indicative of the applicability of the CFVP model in controlling local outbreaks, epidemics, disasters, and future pandemics in rural areas. The broad principles of empowerment of villages, encouragement to volunteerism, and community mobilization can be replicated in emergency situations. Nevertheless, the extent of community engagement and mobilization may be dependent on the perceived susceptibility and magnitude of the specific outbreaks or epidemics in the affected population that was unusually high during an unprecedented epidemic. The sensitization of community stakeholders against the threats of disease and their solutions, rapid estimation of village health needs, and the early deployment of IEC (both traditional and virtual) through community support and mobilization were the cornerstones of CFVP. Building greater trust in the existing public health system and improving service delivery to meet community health needs and expectations can contribute toward accelerating progress toward desired health indicators and outcomes in rural areas of LMICs.

A key feature of CFVP is the application of professionally trained subdistrict (Taluka) coordinators from an NGO acting as the interface between district and the village administration who also supported sensitization and community mobilization effort. When there are no outbreaks, provision of such coordinators will be a constraint due to the associated costs. Future studies should also explore the potential applicability of the CFVP model for long-term impact in strengthening social public health infrastructure, especially human capital, in tackling public health challenges in low-resource rural settings. The applicability of a similar model for health promotion enabling the prevention of high-burden chronic lifestyle diseases, especially diabetes and hypertension, in areas with limited health services warrants further exploration.

### Limitations

There were some significant limitations in the assessment of the COVID-19 vaccination program between Pune and Satara. First, as information on the monthly COVID-19 vaccination statistics was unavailable, we were unable to compare the rates of change in vaccination coverage between both the districts, which precluded the assessment of the effectiveness of the VTFs in accelerating the pace of COVID-19 vaccination in Pune. Second, information on booster dose vaccine utilization was mostly unavailable, as the campaign for the same was initiated toward the end of the study period. Third, there was no available information on vaccine wastage data that could have possibly reduced from CFVP initiatives due to the accurate prior estimation of the beneficiary count in villages preceding vaccine camps. Additionally, the choice of the comparator district was not ideal, but the choice was limited due to factors of administrative and logistic feasibility. For instance, the rural population of Pune comprises 38.9% (3,678,226/9,431,349) of the total population, while the rural population of Satara comprises 81.01% (2,433,031/3,003,741) of the total population. Differences in the extent of spread of infection in rural Pune and Satara, which mediated the extent and quantum of work done to prevent and contain the infection, may also affect the study findings. Finally, the CFVP’s KMS portal was a crucial component of the program, but the utilization of the portal in terms of access and downloading of resources (guidelines, videos, IEC material) stratified by the village stakeholders was not captured during the implementation of CFVP, which prevented assessment of the extent and patterns of its net utilization and its overall usefulness.

### Conclusions

This retrospective impact evaluation of a large-scale rural community–based multipronged intervention for COVID-19 risk reduction and resilience in a single district in western India could not establish a significant change in overall COVID-19 vaccination coverage or reduction in COVID-19 deaths due to the intervention. However, the intervention group showed a significant improvement in knowledge and sustained adherence to COVID-appropriate behaviors. Community-based health interventions engineered by grassroot NGOs and trained volunteers with integration of novel technology and community engagement processes may have considerable applicability in health promotion, protection, and preparedness during future outbreaks and epidemics.

## Appendix

Multimedia Appendix 1 Interventions delivered through the COVID-Free Village Program in Pune in 2021-2022.

Multimedia Appendix 2 Theory of Change Model of the COVID-Free Village Program in Pune in 2021-2022.

Multimedia Appendix 3 Participant interview schedule.

Multimedia Appendix 4 Supplementary data.

## Abbreviations

CFVP: COVID-Free Village Program
CFVS: COVID-Free Village Scheme
IEC: information, education, and communication
KMS: knowledge management system
LMIC: low- and middle-income country
NGO: nongovernmental organization
VTF: village task force

## Data Availability

The data set accompanying this study is available on request to the corresponding author.
