# Supplementary material for: COVID-19 Resilience and Risk Reduction Intervention in Rural Populations of Western India: Retrospective Evaluation
Source: JMIR Public Health Surveill. 2024 Jul 29;10:e47520. doi: 10.2196/47520 (PMC11319881; doi:10.2196/47520)
Supplement: Multimedia Appendix 1 [file publichealth_v10i1e47520_app1.pdf]

INTERVENTION

Conduct meetings with VTFs, older adults, social workers, and self-motivated persons to discuss and finalize the activities to be conducted in the village based on their felt needs

All the activities in the village are guided & supported by **Sarpanch & Gramsevak in support with Taluka Coordinators under the guidance of District Coordinators**

Members of VTFs, older adults, self-motivated persons, social workers, non-government organizations play important role the activities toward COVID free village initiative

VTF-1

Awareness and Covid appropriate behavior

- Conduct home visits
- IEC (TV/WhatsApp/Social Media)
- Sanitation of common places/streets
- Distribution of masks and sanitizers
- Finding local sponsors and donors

Covid Help Center

- Provide village-level key information on Covid status
- Disseminate positive messages
- Make emergency transportation
- Emergency contact information

Potential risk/challenges:

- People may not have TV, mobile phones, or any media exposure
- Limited funding

VTF-2

Trace, Track, Test and Treat

- Support ASHA workers in surveillance
- Facilitate referrals for Covid testing
- Help in tracing contacts of suspects
- Counsel villagers on measures to be taken if tested positive for COVID-19

Quarantine Centers (QC) & Covid Care Centers (CCC)

- Help in identifying patients who needed admission in QC/CCC and facilitate the admission
- Support for setting-up of the Quarantine Center/Covid Care Center

Task forces to work in accordance with local requirements. If and when a rise in new infections occur, the appropriate VTF is activated as per the need, and when the number

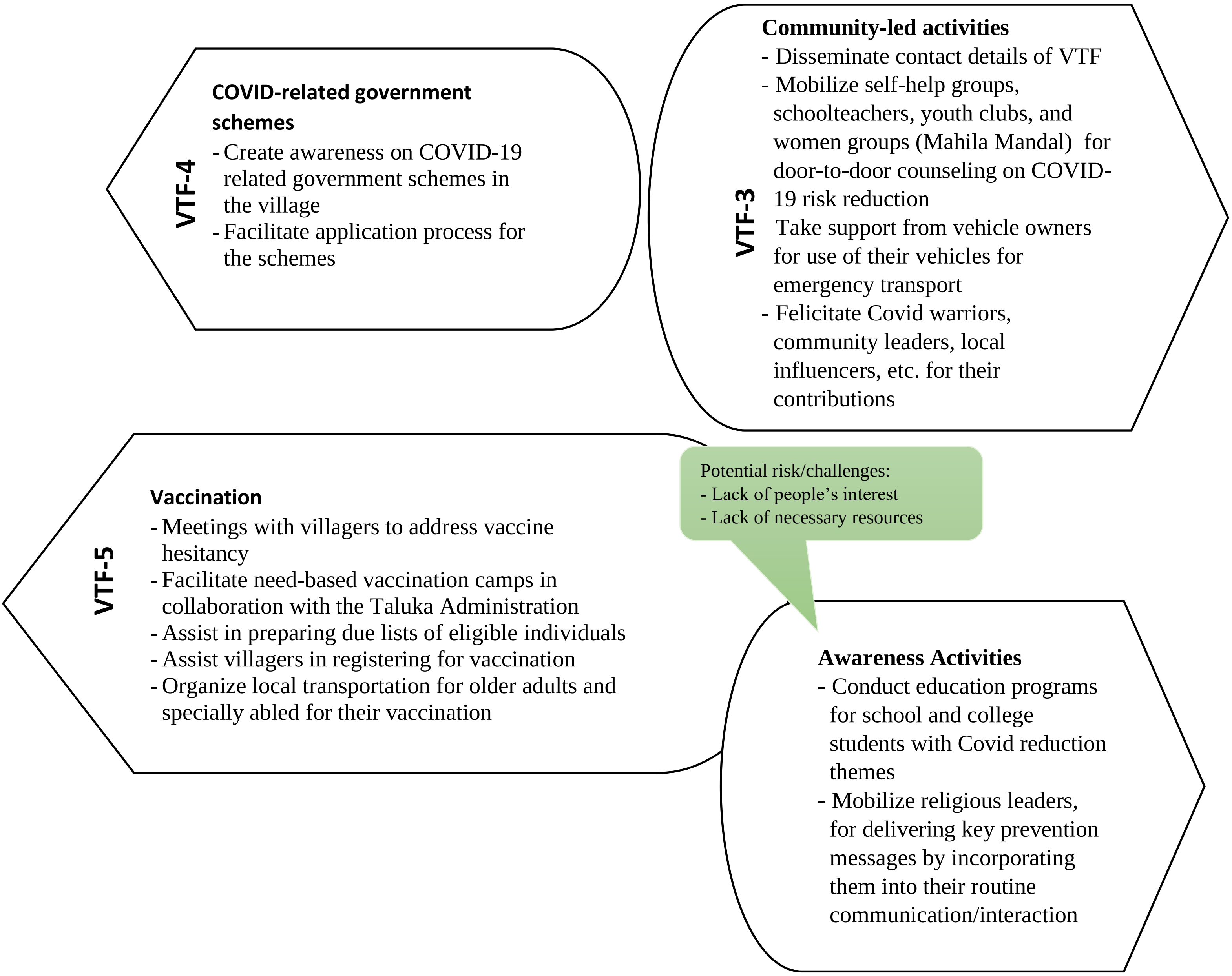
VTF-4
COVID-related government schemes
- Create awareness on COVID-19 related government schemes in the village
- Facilitate application process for the schemes
VTF-3
Community-led activities
- Disseminate contact details of VTF
- Mobilize self-help groups, schoolteachers, youth clubs, and women groups (Mahila Mandal) for door-to-door counseling on COVID-19 risk reduction
Take support from vehicle owners for use of their vehicles for emergency transport
- Felicitate Covid warriors, community leaders, local influencers, etc. for their contributions
VTF-5
Vaccination
- Meetings with villagers to address vaccine hesitancy
- Facilitate need-based vaccination camps in collaboration with the Taluka Administration
- Assist in preparing due lists of eligible individuals
- Assist villagers in registering for vaccination
- Organize local transportation for older adults and specially abled for their vaccination
Potential risk/challenges:
- Lack of people's interest
- Lack of necessary resources
Awareness Activities
- Conduct education programs for school and college students with Covid reduction themes
- Mobilize religious leaders, for delivering key prevention messages by incorporating them into their routine communication/interaction
