# Supplementary material for: COVID-19 Resilience and Risk Reduction Intervention in Rural Populations of Western India: Retrospective Evaluation
Source: JMIR Public Health Surveill. 2024 Jul 29;10:e47520. doi: 10.2196/47520 (PMC11319881; doi:10.2196/47520)
Supplement: Multimedia Appendix 2 [file publichealth_v10i1e47520_app2.pdf]

COVID-Free Village Program (CFVP) Theory of Change

COVID Free Village

Inputs- BJS

- Program Frameworks, processes, SOPs, guidelines, audio visuals, guidebooks, tools
- Training and capacity building of University Faculties, NSS students, ANMs/ASHAs, Sarpanch & VTFs
- Program Management Platform
- Partnerships for implementation, capacity building, monitoring, research, knowledge and funding

Actions- Community

- Enabling environment for risk reduction
- Mobilization of local resources
- COVID appropriate behavior
- Linkages with district/ block administration

Support- State Government

- Directive for implementation as government scheme
- Mobilization university faculties, NSS network, frontline health workers (ANMs/ASHAs) for capacity building and data reporting
- COVID-free village competitions & prizes
- District/ block administration support for government schemes, vaccines etc.

PROCESSES

Adaptive Capacities

Enabling village/community for planning, decision making, implementation and assessment of preparedness and response

Provision of public information on preparedness, prevention and relevant strategies/ actions

Absorptive & Transformative Capacities

Mobilization and linkages with local government/ stakeholders and existing village/ community-based organizations for effective response and actions and provide integrated care/ support to the community (eg. Tracing, Tracking, Testing & Treatment, vaccination, QCs/CCCs etc.)

Transformative Capacities

Encouraging the village/ communities to adopt and maintain COVID-appropriate behaviors, and health monitoring & seeking behaviors through awareness & education, and enabling them to build their resilience

Target strategies to engage and support vulnerable population and community members for access to health, social and economic services & systems

OUTPUTS

- No. of Sarpanch & VTFs attending training on CFV
- Increase in number of villages participating in the CFV
- No. of villagers conducting COVID awareness and CAB activities
- Increased village/ community involvement in prevention, treatment and support services
- Increased COVID tracking and testing in villages as per need
- Increased referral of COVID positive cases to the CCCs and hospitals
- No. of villages establishing health infra (QCs/CCCs) and mobilizing health care resources/ services (ANMs/ASHAs)
- Eligible villagers receive benefits of government schemes
- Increased uptake of COVID vaccination in

PHASE- I OUTCOMES

Demand-responsiveness of village/ community for CFV

- Sarpanch ownership towards CFV
- VTF formation by community
- Participation in capacity building measures
- Participation in VTF activities
- Ownership of VTFs towards CFV

Augmentation of government efforts through VTFs

Preparedness & Support
- Community awareness on CAB
- Awareness & support for utilization of government schemes

Prevention & Support
- COVID Prevention through universal vaccination coverage

Containment & Support
- Need-based containment measures through community mobilization

Behavior change in village/community enabled by CFV

- Sense of agency to make village pandemic resilient
- Reduced vaccination hesitancy
- Improved adoption of CAB
- Increase trust in government administration for seeking support

COVID resilient Village

Towards CFV ownership & strengthening resilience capacity

Towards resilience & enabling effective resilience response

GOVERNMENT OWNERSHIP

COMMUNITY LEADERSHIP

ENABLERS

- **Tech-based universal access to resource material; capacity building and monitoring support systems**
- **Support by BJS Volunteer network**

ASSUMPTIONS

- **Community-demand for program sustained over pandemic life-cycle**
- **Government policies remain supportive of program implementation**
- **COVID-free Village competitions declared to motivate village participation**
- **NSS network available for capacity building and handholding in villages**
- **ASHA and ANM network available for data reporting**

FACTORS THAT AFFECT PROCESSES & OUTCOMES

Environment- Decrease in COVID severity, decrease in number of COVID cases
Community- Community willingness & involvement, available resources/ infrastructure, administration & health system support
Individual- Knowledge, attitudes, behavior, motivation, social support, access to resources and ability to implement strategies

ACRONYMS

**ANM**	Auxiliary Nurse Midwife
**ASHA**	Accredited Social Health Activist
**BJS**	Bhartiya Jain Sanghatana
**CAB**	COVID Appropriate Behavior
**CCC**	COVID Care Centre
**CFV**	COVID Free Village
**NSS**	National Service Scheme
**QC**	Quarantine Centre
**VTF**	Village Task Force

DEFINITIONS

***Adaptive Capacity*:** The ability to make proactive and informed choices regarding preparedness and response by applying information/ knowledge and skills received through training and capacity building

***Absorptive Capacity*:** The ability of individuals, households, communities or systems to collectively minimize the COVID risk and burden and recover from it through testing, treatment, vaccination and relief and support measures

***Transformative Capacity*:** The ability to create an enabling environment through awareness and education, COVID appropriate behavior, utilization of village resources/ infrastructure, relief/ service support and delivery and policies/ regulations that constitute the guidelines/ conditions necessary for change and resilience

***Civic Mindedness and social responsibility*:** The ability of people and community to do right things individually and collectively by adopting good and appropriate behavior practices, and a sense of responsibility towards one's community, to each other and the society in order to build the resilience

Individual Behavior & Social Consciousness: The ability of individuals to adopt right behavior and to understand what good they need to do for themselves, and the community to build resilience
