# Supplementary material for: COVID-19 Resilience and Risk Reduction Intervention in Rural Populations of Western India: Retrospective Evaluation
Source: JMIR Public Health Surveill. 2024 Jul 29;10:e47520. doi: 10.2196/47520 (PMC11319881; doi:10.2196/47520)
Supplement: Multimedia Appendix 3 [file publichealth_v10i1e47520_app3.pdf]

Assessment of Covid Free Village Program (CFV) for Covid-19 Risk Reduction
कोविड मुक्त गाव (CFV) कार्यक्रमाचे मूल्यांकन

HOUSEHOLD SURVEY TOOL
घरगुती सर्वेक्षण साधन

I. IDENTIFICATION- ओळख

**DATE दिनांक**	
**DISTRICT ID जिल्हा ID**	
**TALUKA ID तालुका ID**	
**VILLAGE ID गावाचा ID**	
**HOUSEHOLD ID घराचा ID**	
**UNIQUE ID विशेष ID**	
**NAME OF THE INTERVIEWER**<br>**मुलाखत घेणाऱ्याचे नाव**	
**INTERVIEW START TIME:**<br>**मुलाखत प्रारंभ वेळ**	**INTERVIEW END TIME:**<br>**मुलाखत समाप्त वेळ**
**NAME OF THE RESPONDENT**	
**Contact No. संपर्क क्र.**<br>(If HH don't have phone/ any contact number then write 9999999999)	
**Address पत्ता**	

II. SOCIODEMOGRAPHIC INFORMATION- सामाजिक-लोकसंख्याशास्त्र विषयक माहिती

Respondent Category	A. Head of Household<br>B. Other Informed Family Member
1.1 Name of head of household/ member<br>कुटुंबप्रमुखाचे नाव	……….... ……………. …………..<br>First name Middle name Last name<br>पहिले नाव मधले नाव आडनाव
1.2 Age of the head of the household/ member<br>कुटुंबप्रमुखाचे वय	
1.3 Sex of the head of the household/ member<br>कुटुंबप्रमुखाचे लिंग	1. Male पुरुष<br>2. Female स्त्री<br>3. Other इतर
1.4 Religion of the head of household/ member<br>कुटुंबप्रमुखाचा धर्म	1. Hindu हिंदू<br>2. Muslim मुस्लिम<br>3. Buddhist बौद्ध<br>4. Christian ख्रिश्चन<br>5. Sikh शीख<br>6. Jain जैन<br>7. Other/Prefer not to say इतर/सांगू इच्छित नाही
1.5 Caste of the head of household/ member<br>कुटुंबप्रमुखाची जात	1. OBC इतर मागास वर्ग<br>2. SC अनुसूचित जाती

	3. ST अनुसूचित जमाती<br>4. General (Open) सामान्य (खुला)<br>5. Other/Prefer not to say इतर/सांगू इच्छित नाही
1.6. Education status – head of household / member<br><br>कुटुंबप्रमुखाची शैक्षणिक पात्रता	1. Illiterate अशिक्षित<br>2. Primary school certificate प्राथमिक शिक्षण प्रमाणपत्र<br>3. Middle school certificate माध्यमिक शिक्षण प्रमाणपत्र<br>4. High school (Matric) certificate उच्च माध्यमिक शिक्षण (मॅट्रिक) प्रमाणपत्र<br>5. Intermediate or diploma अंत:स्थ किंवा पदविका<br>6. Graduate पदवीधर<br>7. Post-Graduate पदव्युत्तर<br>8. Professional व्यावसायिक
1.6.1 Educational status – member with highest educational qualification (if different from head of household/ member)<br><br>सर्वाधिक शैक्षणिक पात्रता असलेला सदस्य (कुटुंबप्रमुखापेक्षा पात्रता जास्त/ वेगळी असल्यास)	1. Illiterate अशिक्षित<br>2. Primary school certificate प्राथमिक शिक्षण प्रमाणपत्र<br>3. Middle school certificate माध्यमिक शिक्षण प्रमाणपत्र<br>4. High school (Matric) certificate उच्च माध्यमिक शिक्षण (मॅट्रिक) प्रमाणपत्र<br>5. Intermediate or diploma अंत:स्थ किंवा पदविका<br>6. Graduate पदवीधर<br>7. Post-Graduate पदव्युत्तर<br>8. Professional व्यावसायिक
1.6.2 Sex of the member with highest educational qualification<br>सर्वाधिक शैक्षणिक पात्रता असलेल्या सदस्याचे लिंग	1. Male पुरुष<br>2. Female स्त्री<br>3. Other इतर
1.7 Employment status of the head of household/ member<br><br>कुटुंबप्रमुखाच्या रोजगाराची स्थिती	1. Unemployed बेरोजगार<br>2. Unskilled agricultural or craft worker अकुशल शेती कामगार किंवा कारागीर<br>3. Skilled agricultural or craft worker कुशल शेती कामगार किंवा कारागीर<br>4. Business / Shop owner व्यापारी / दुकानदार<br>5. Professional व्यावसायिक<br>6. Agriculture शेती<br>7. Government Service सरकारी नोकरी<br>8. Private Job खाजगी नोकरी<br>9. Housewife<br>10. Other इतर
1.8 Is any household member a healthcare worker?	1. Yes 2. No

(ASHA/ANM/Nurse/Ayush doctor etc)<br>कुटुंबातील कोणी व्यक्ति आरोग्यसेवेत आहे का?<br>(ASHA/ANM/नर्स /आयुष डॉक्टर इ.)	१. होय २. नाही
1.9 Household type<br>कुटुंबाचा प्रकार	1. Joint 2. Nuclear<br>१. एकत्र २. विभक्त
1.9.1 Total number of rooms in the household<br>घरातील एकूण खोल्यांची संख्या	
1.9.2 Total number of household members?<br>कुटुंबातील सदस्यांची संख्या	
1.9.3 Household composition कुटुंबाची रचना<br>• Elderly (60+) ज्येष्ठ<br>• Children (6-18) मुले<br>• Under-5 children पाच वर्षांखालील मुले<br>• Pregnant women गर्भवती महिला	 1. Yes 2. No १.हो २.नाही<br>1. Yes 2. No १.हो २.नाही<br>1. Yes 2. No १.हो २.नाही<br>1. Yes 2. No १.हो २.नाही
1.10 Is there a functional toilet in the household?<br>घरात चालू स्थितीतील शौचालय आहे का?	1. Present 2. Absent<br>१.आहे २.नाही
1.11 Comorbidities amongst household members<br>कुटुंबातील सदस्यांना सहव्याधी (इतर आजार) आहेत का?<br>• Diabetes मधुमेह<br>• Hypertension उच्च रक्तदाब<br>• Heart Disease हृदयविकार<br>• Cancer कर्करोग<br>• Chronic respiratory illness दीर्घकालीन श्वसनरोग	 <br>1. Present 2. Absent १.आहे २.नाही<br>1. Present 2. Absent १.आहे २.नाही<br>1. Present 2. Absent १.आहे २.नाही<br>1. Present 2. Absent १.आहे २.नाही<br>1. Present 2. Absent १.आहे २.नाही
1.12 Are you aware of Village level committee or Taskforces formed at the village level for Covid prevention & management?<br>कोविड नियंत्रण आणि व्यवस्थापनासाठी गाव पातळीवर स्थापन करण्यात आलेल्या ग्रामस्तरीय समिती किंवा ग्राम कृती दलांबद्दल/ पथकांबद्दल (टास्कफोर्सबद्दल) तुम्हाला माहिती आहे का?	1. Yes 2. No 3. Not aware<br>१.हो २.नाही ३. माहीत नाही
1.12.1 Are you or any members from your household is member of Village level committee or Taskforces formed at the village level for Covid prevention & management?<br>आपण किंवा आपल्या घरातील कोणीही सदस्य कोविड नियंत्रण आणि व्यवस्थापनासाठी गाव पातळीवर तयार केलेल्या ग्रामस्तरीय समितीचे किंवा ग्राम कृती दलांचे/ पथकांचे (टास्कफोर्सचे) सदस्य आहात का?	1. Yes 2. No 3. NA<br><br>१.हो २.नाही ३. लागू नाही

III. HOUSEHOLD COVID-19 VACCINATION STATUS कुटुंबाच्या कोविड-१९ लसीकरणाची स्थिती

3.1 Total number of household members aged ≥12 years (eligible for vaccination)<br>कुटुंबातील १२ वर्षांपेक्षा जास्त वय असलेले सदस्य (लसीकरणास पात्र)	

<table>
<tr>
<td>3.2 Vaccination status of household members eligible for Covid-19 vaccination
कोविड -१९ लसीकरणास पात्र असलेल्या कुटुंबातील सदस्यांच्या लसीकरणाची स्थिती
3.2.1 Age वय
3.2.2 Sex लिंग
3.2.3 Total doses received (0,1,2,3)
मिळालेले एकूण डोस (०,१,२,३)
3.2.4 Date of receiving first dose
पहिला डोस मिळाल्याची तारीख/ महिना
3.2.5 Date of receiving second dose
दुसरा डोस मिळाल्याची तारीख/ महिना
3.2.6 Date of receiving booster dose
बुस्टर डोस मिळाल्याची तारीख/ महिना
3.2.7 Whether vaccination verified from certificate or from recall
लसीकरणाची प्रमाणपत्राद्वारे किंवा स्मरणाद्वारे पडताळणी</td>
<td>
<table>
<tr><th>सदस्य</th><th>१</th><th>२</th><th>३</th><th>४</th><th>५</th></tr>
<tr><td>वय</td><td></td><td></td><td></td><td></td><td></td></tr>
<tr><td>लिंग</td><td></td><td></td><td></td><td></td><td></td></tr>
<tr><td>एकूण डोस</td><td></td><td></td><td></td><td></td><td></td></tr>
<tr><td>पहिला डोस
तारीख/ महिना</td><td></td><td></td><td></td><td></td><td></td></tr>
<tr><td>दुसरा डोस
तारीख/ महिना</td><td></td><td></td><td></td><td></td><td></td></tr>
<tr><td>बुस्टर डोस
तारीख/ महिना</td><td></td><td></td><td></td><td></td><td></td></tr>
<tr><td>पडताळणी/
स्मरण</td><td></td><td></td><td></td><td></td><td></td></tr>
</table>
</td>
</tr>
<tr>
<td>3.3 Site of initial vaccination of elderly/comorbid household members
कुटुंबातील ज्येष्ठ/ सहव्याधी (इतर आजार) असलेल्या सदस्यांच्या सुरुवातीच्या लसीकरणाचे ठिकाण</td>
<td>1. Government facility सरकारी सुविधा
2. Private facility खाजगी सुविधा
3. Government vaccination camp
सरकारी लसीकरण शिबिर
4. Not Applicable लागू नाही</td>
</tr>
<tr>
<td>3.4 Site of initial vaccination of other household members
(Multi select- ask verbatim)
कुटुंबातील इतर सदस्यांच्या सुरुवातीच्या लसीकरणाचे ठिकाण
(बहुपर्यायी - मुलाखातदाराने दिलेल्या उत्तरानुसार दिलेल्या पर्यायांपैकी फक्त एक पर्याय सेलेक्ट करा)
(If vaccination at Govt. facility or Govt. vaccination camp, go to Q 3.5)
(जर लसीकरण सरकारी सुविधा किंवा सरकारी लसीकरण शिबिरामध्ये झाले असेल तर प्र. ३.५ ला जावे)</td>
<td>1. Government facility सरकारी सुविधा
2. Private facility खाजगी सुविधा
3. Government vaccination camp
सरकारी लसीकरण शिबिर
4. Household vaccination घरोघरी लसीकरण</td>
</tr>
<tr>
<td>3.4.1 If vaccination was done at Private facility, was there any cost incurred in getting vaccination?
जर खाजगी सुविधेत लसीकरण केले गेले, तर लसीकरण करण्यासाठी काही खर्च आला होता का?</td>
<td>1. Yes 2. No १.हो २.नाही
(If No, go to Q 3.5) (जर नाही, तर प्र. ३.५ ला जावे)</td>
</tr>
<tr>
<td>3.4.2 If Yes, how much money was spent for vaccination?
जर हो, तर लसीकरणासाठी किती पैसे खर्च केले गेले?</td>
<td>_____ (in INR)
_____ (भारतीय रुपयांमध्ये)</td>
</tr>
<tr>
<td>3.5 What difficulties/ challenges you faced for the vaccination at vaccination sites?
(Multi select- ask verbatim)</td>
<td>1. Non-availability of the vaccine लस उपलब्ध नसणे
2. Long ques/ delay/ rush for the vaccination
लसीकरणासाठी लांबलचक रांग/ विलंब/ गर्दी
3. Unavailability of expected vaccine</td>
</tr>
</table>

लसीकरणाच्या ठिकाणी लसीकरणासाठी तुम्हाला कोणत्या अडचणी/ आव्हानांचा सामना करावा लागला?<br>(बहुपर्यायी - मुलाखातदाराने दिलेल्या उत्तरानुसार दिलेल्या पर्यायांपैकी फक्त एक पर्याय सेलेक्ट करा)	अपेक्षित लसीची अनुपलब्धता<br>4. Other इतर<br>5. No difficulties/ challenges faced<br>कोणत्याही अडचणी/ आव्हाने नाहीत
3.6 What was the distance of the vaccination site from your household?<br>***(If the distance is less than 1 km, skip Q.3.7)***<br>तुमच्या घरापासून लसीकरणाच्या ठिकाणापर्यंतचे अंतर किती होते?<br>***(जर अंतर 1 किमीपेक्षा कमी असेल तर पुढील प्रश्न 3.7 वगळा)***	1. < 1 km < १ किमी<br>2. 2-3 km २-३ किमी<br>3. 4-5 km ४-५ किमी<br>4. >5 km > ५ किमी<br>5. Outside the village गावाबाहेर
3.7 Were you or any HH member or anyone from the village provided with any transport / pick and drop facility for Covid-19 vaccination since………month?<br>...........महिन्यापासून कोविड-१९ लसीकरणाकरिता तुमच्यासाठी किंवा तुमच्या घरातील एखाद्या सदस्यासाठी किंवा गावातील एखाद्या व्यक्तीसाठी वाहतुकीची/ ने-आणीची काही सोय करण्यात आली होती का?	1. Yes १. हो<br>2. No २. नाही
3.8 Reasons for unvaccinated status (1st/2nd dose) in any household member<br>(Go to 3.9 if all vaccinated)<br>(Multi select- ask verbatim)<br><br>कुटुंबातील एखाद्या सदस्याचे लसीकरण झालेले नसल्याच्या स्थितीची कारणे (पहिला/दुसरा डोस)<br>(सर्वांचे लसीकरण झाले असल्यास प्र. ३.९ ला जावे)<br>(बहुपर्यायी - मुलाखातदाराने दिलेल्या उत्तरानुसार दिलेल्या पर्यायांपैकी फक्त एक पर्याय सेलेक्ट करा)	1. Have concern over side effects<br>अनिष्ट परिणामाची भीती<br>2. Have concerns over efficacy of vaccine<br>लसीच्या परिणामकारकतेविषयी शंका<br>3. Vaccine is not always available<br>लस नेहमी उपलब्ध नसणे<br>4. Unable to access the vaccine site<br>लसीकरणाच्या संकेतस्थळात प्रवेश करू न शकणे<br>5. Medical contraindications<br>विपरीत परिणामाविषयीचा वैद्यकीय इशारा<br>6. Pregnant Women गर्भवती महिला<br>7. No specific reason काही विशिष्ट कारण नाही<br>8. Not Applicable लागू नाही
3.9 Did you come across Covid-19 vaccination awareness related home-visit since the month of _______?<br>______ महिन्यापासून तुम्ही कोविड-१९ लसीकरण जागरुकतेसंबंधी गृहभेट अनुभवली आहे का?	1. Yes १. हो<br>2. No २. नाही
3.10 What are your thoughts/ perceptions regarding Covid vaccine?<br>(multiple response permitted - query verbatim)<br><br>कोविड लसीबाबत तुमचे विचार/ समज काय आहेत?<br><br>(बहुपर्यायी- मुलाखातदाराने दिलेल्या उत्तरातून विविध पर्यायांना सेलेक्ट करा)	1. Vaccine is good लस चांगली आहे<br>2. Increases immunity प्रतिकरशक्ती वाढते<br>3. Prevents severity of Covid<br>गंभीर कोविड होण्यापासून रोखते<br>4. Less chances of death मृत्युची कमी शक्यता<br>5. Less risk for other HH members<br>इतर कुटुंबातील सदस्यांना कमी धोका<br>6. Side-effects of the vaccine लसीमुळे इतर दुष्परिणाम<br>7. Decreases immunity प्रतिकरशक्ती कमी होते

	8. No use of vaccines लसीचा काही फायदा नाही<br>9. Other इतर

IV. PREVENTION- प्रतिबंध

4.1 What measures are you aware of for the prevention of Covid-19?<br>(multiple response permitted – query verbatim)<br><br>कोविड-१९ प्रतिबंधाकरिता असलेल्या कोणत्या उपायांविषयी तुम्हाला माहिती आहे?<br><br>(बहुपर्यायी- मुलाखातदाराने दिलेल्या उत्तरातून विविध पर्यायांना सेलेक्ट करा)	1. Wearing a mask outside<br>बाहेर जाताना मास्क लावणे<br>2. Maintaining distance अंतर पाळणे<br>3. Handwashing with soap / sanitizer<br>साबण/ सॅनिटायझरने हात धुणे<br>4. Taking immunity boosting drug<br>प्रतिकारशक्ति वाढविणारे औषध घेणे<br>5. Vaccination लसीकरण<br>6. None कोणतेही नाही
4.2 Covid-19 disease can be a serious illness?<br><br>कोविड-१९ हा गंभीर रोग ठरू शकतो का?	1. Strongly Agree एकदम सहमत आहे<br>2. Agree सहमत आहे<br>3. Neutral तटस्थ<br>4. Disagree सहमत नाही<br>5. Strongly Disagree अजिबात सहमत नाही
4.3 Covid-19 disease is more serious in which population groups?<br>(multiple response permitted – query verbatim)<br><br>कोणत्या लोकांना कोविड-१९ रोग होण्याचा धोका जास्त आहे?<br><br>(बहुपर्यायी- मुलाखातदाराने दिलेल्या उत्तरातून विविध पर्यायांना सेलेक्ट करा)	1. Elderly ज्येष्ठ<br>2. Heart patients हृदयरोगी<br>3. Diabetes मधुमेह<br>4. Hypertension patients उच्च रक्तदाबाचे रुग्ण<br>5. Cancer patients कर्करोगाचे रुग्ण<br>6. Lung disease / Asthma फुफ्फुस रोग/ दमा<br>7. Children मुले<br>8. Pregnant women गर्भवती महिला<br>9. Weak/ People with Less immunity<br>अशक्त/ कमी प्रतिकरशक्ती असलेले<br>10. Don’t know माहित नाही
4.4. Are you aware of any Covid-19 variants?<br>(multiple response permitted – query verbatim)<br><br>तुम्हाला कोविड-१९ च्या निरनिराळ्या स्वरूपांविषयी माहिती आहे का? (बहुपर्यायी- मुलाखातदाराने दिलेल्या उत्तरातून विविध पर्यायांना सेलेक्ट करा)	1. Alpha अल्फा<br>2. Delta डेल्टा<br>3. Omicron ओमीक्रॉन<br>4. Don’t know माहित नाही
4.5 Have you come across Covid-19 awareness campaigns in your village?<br>तुम्हाला तुमच्या गावात कोविड-१९ बाबत जागरुकता करताना/ माहिती देताना लोक आढळले का?	1. Yes हो<br>2. No नाही<br>***(If No, go to Q 4.6) (जर नाही, तर प्र. ४.६ ला जावे)***
4.5.1 If Yes, what was the subject/ focus of awareness campaigns?<br>(multiple response permitted – query verbatim)<br><br>जर हो, तर जनजागृती मोहिमेचा विषय काय होता?	1. Covid Testing कोविड चाचणी (टेस्टिंग)<br>2. Covid Vaccination कोविड लसीकरण<br>3. Nutrition & Medicines पोषण आणि औषधे<br>4. Covid-Appropriate Behaviour कोविड-योग्य वर्तन<br>5. Covid-related Government Schemes

(बहुपर्यायी- मुलाखातदाराने दिलेल्या उत्तरातून विविध पर्यायांना सेलेक्ट करा)	कोविड संबंधित सरकारी योजना<br>6. Other इतर<br>7. Don't Know/ Can't Say<br>माहित नाही/ सांगू शकत नाही
4.5.2 What methods of Covid-19 awareness have been experienced by you or any member of your household?<br>(multiple response permitted – query verbatim)<br><br>तुमच्या कुटुंबातील एखाद्या सदस्याने कोणत्या कोविड-१९ जागरूकता पद्धती अनुभवल्या आहेत?<br>(बहुपर्यायी- मुलाखातदाराने दिलेल्या उत्तरातून विविध पर्यायांना सेलेक्ट करा)	1. Posters पोस्टर्स<br>2. Street Play पथनाट्य<br>3. Public Announcement जाहीर घोषणा<br>4. Influential People प्रभावी लोक<br>5. Religious Leaders धार्मिक नेते<br>6. Kirtan किर्तन<br>7. Prabhat Feri प्रभात फेरी<br>8. Bachat Gat बचत गट<br>9. Tarun Mandal तरुण मंडळ<br>10. Home Visits गृहभेटी<br>11. Social Media (WhatsApp, Facebook etc.)<br>सामाजिक माध्यमे (व्हाट्सअप, फेसबुक इ.)<br>12. TV/Radio/Newspaper टीव्ही/ रेडिओ/ वर्तमानपत्र<br>13. Other इतर
4.6 How often do you wash your hands with soap/ sanitizer?<br><br>तुम्ही किती वेळा साबणाने/ सॅनिटायझरने हात धुता?	1. Always (~6 or more times)<br>नेहमी (६ वेळा किंवा ६ पेक्षा जास्त)<br>2. Mostly (~4-5 times) बऱ्याच वेळा (~४-५ वेळा)<br>3. Occasionally (~3 times) अधूनमधून (~३ वेळा)<br>4. Rarely (~2 or less times)<br>क्वचित (२ वेळा किंवा २ पेक्षा कमी)<br>5. Never कधीही नाही
4.7 How often do you wear masks while going out of home?<br><br>घराबाहेर जाताना तुम्ही किती वेळा मास्क घालता?	1. Always (~6 or more times)<br>नेहमी (६ वेळा किंवा ६ पेक्षा जास्त)<br>2. Mostly (~4-5 times) बऱ्याच वेळा (~४-५ वेळा)<br>3. Occasionally (~3 times) अधूनमधून (~३ वेळा)<br>4. Rarely (~2 or less times)<br>क्वचित (२ वेळा किंवा २ पेक्षा कमी)<br>5. Never कधीही नाही
4.8 Does your family have any difficulty in access or affording soap for handwashing?<br>तुमच्या कुटुंबाला साबण मिळविणे किंवा खरेदी करणे यांत काही अडचण आहे का?	1. Yes, often हो, नेहमी<br>2. Yes, sometimes हो, कधी कधी<br>3. No नाही
4.9 Does your family have any difficulty in access or affording sanitizer for hand hygiene?<br>तुमच्या कुटुंबाला सॅनिटायझर मिळवण्यामध्ये किंवा खरेदी करण्यामध्ये काही अडचण आहे का?	1. Yes, often हो, नेहमी<br>2. Yes, sometimes हो, कधी कधी<br>3. No नाही
4.10 Does your family have any difficulty in access or affording masks?<br>तुमच्या कुटुंबाला मास्क मिळविणे किंवा खरेदी करणे यांत काही अडचण आहे का?	1. Yes, often हो, नेहमी<br>2. Yes, sometimes हो, कधी कधी<br>3. No नाही
4.11 Has tobacco smoking by any of the	1. Yes, reduced significantly

household members changed since intervention / month?<br><br>______ महिन्यापासून कुटुंबातील एखाद्या सदस्याच्या धूम्रपान करण्यामध्ये (सिगरेट ओढणे/ फुकणे) बदल झाला आहे का?	हो, मोठ्या प्रमाणात कमी झाले<br>2. Yes, reduced mildly<br>हो, काही प्रमाणात कमी झाले<br>3. No change काही बदल नाही<br>4. Yes, increased significantly<br>हो, मोठ्या प्रमाणात वाढ झाली<br>5. Yes, increased mildly<br>हो, काही प्रमाणात वाढ झाली<br>6. No smoker in the household<br>घरामध्ये धूम्रपान करणारे (सिगरेट फुकणारे) कुणीही नाही<br>7. Don't know माहीत नाही
4.12 Has smokeless tobacco use by any of the household members changed since intervention / month?<br><br>______ महिन्यापासून कुटुंबातील एखाद्या सदस्याच्या तंबाखूच्या धूम्रपानामध्ये (सेवनामध्ये) बदल झाला आहे का?	1. Yes, reduced significantly<br>हो, मोठ्या प्रमाणात कमी झाले<br>2. Yes, reduced mildly<br>हो, काही प्रमाणात कमी झाले<br>3. No change काही बदल नाही<br>4. Yes, increased significantly<br>हो, मोठ्या प्रमाणात वाढ झाली<br>5. Yes, increased mildly<br>हो, काही प्रमाणात वाढ झाली<br>6. No smokeless tobacco user in the household<br>घरामध्ये तंबाखू खाणारे/ सेवन करणारे कुणीही नाही<br>7. Don't know माहीत नाही
4.13 Has alcohol use by any of the household members changed since intervention / month?<br><br>______ महिन्यापासून कुटुंबातील एखाद्या सदस्याच्या दारू/ मद्यपेय घेण्यामध्ये (पिण्यामध्ये) बदल झाला आहे का?	1. Yes, reduced significantly<br>हो, मोठ्या प्रमाणात कमी झाले<br>2. Yes, reduced mildly<br>हो, काही प्रमाणात कमी झाले<br>3. No change काही बदल नाही<br>4. Yes, increased significantly<br>हो, मोठ्या प्रमाणात वाढ झाली<br>5. Yes, increased mildly<br>हो, काही प्रमाणात वाढ झाली<br>6. No alcohol user in the household<br>घरामध्ये दारू/ मद्यपेय घेणारे/ पिणारे कुणीही नाही<br>7. Don't know माहीत नाही
4.14 Has any family member ever installed Aarogya Setu application on their smartphone?<br>तुमच्या कुटुंबातील एखाद्या सदस्याने त्याच्या/ तिच्या स्मार्टफोनवर आरोग्य सेतु ॲप स्थापित (इंस्टॉल) केले आहे का ?	1. Yes हो<br>2. No नाही<br>3. Don’t have smartphone आमच्याकडे स्मार्टफोन नाही<br>***(If No OR Don’t have smartphone, go to Q 5.1)***<br>***(जर नाही किंवा स्मार्टफोन नाही, तर प्र. ५.१ ला जावे)***
4.14.1 If Yes, for what purpose Aarogya Setu application was used/ installed?<br><br>जर हो, तर आरोग्य सेतू ॲप कोणत्या उद्देशाने वापर / स्थापित	1. Identify potential hotspots/ contact tracing<br>संभाव्य हॉटस्पॉट्स / कॉन्टॅक्ट ट्रेसिंग ओळखण्यासाठी<br>2. Self-Assessment Test<br>स्वताची चाचणी (टेस्ट) करण्यासाठी<br>3. Understand risk of infection status

केले गेले?<br><br>(Query Verbatim and multi-select)<br><br>(बहुपर्यायी- मुलाखातदाराने दिलेल्या उत्तरातून विविध पर्यायांना सेलेक्ट करा)	संसर्गाच्या स्थितीचा धोका समजून घेण्यासाठी<br>4. Know about Covid-19 test status<br>कोविड -19 चाचणी स्थितीबद्दल जाणून घेण्यासाठी<br>5. Understand hygiene and social distancing protocols<br>स्वच्छता आणि सामाजिक अंतराचे नियम समजून घेण्यासाठी<br>6. Apply/ Get E-pass<br>अर्ज करण्यासाठी/ ई-पास मिळवण्यासाठी<br>7. Get Updates, advisory and best practices related to COVID-19<br>कोविड -19 शी संबंधित अद्यावत माहिती, सूचना आणि सर्वोत्तम पद्धती जाणून घेण्यासाठी<br>8. Get List of COVID-19 testing facilities and ICMR approved Labs<br>कोविड -19 चाचणी सुविधा आणि आयसीएमआरने मंजूर केलेल्या लॅबची यादी मिळवण्यासाठी<br>9. Get Emergency Helpline contacts<br>आपात्कालीन हेल्पलाईन संपर्क साधण्यासाठी<br>10. Other इतर<br>11. None कोणत्याही नाही

V. PREPAREDNESS- सज्जता

5.1 Has any member of your household been ever tested (Antigen/RTPCR) for Covid-19?<br>तुमच्या कुटुंबातील एखाद्या सदस्याची कोविड-१९ (कोरोना) चाचणी (अँटीजेन/ आरटीपीसीआर टेस्ट) झाली आहे का?	1. Yes 2. No १. हो २ नाही.<br>***(If No, go to Q 5.4) (जर नाही, तर प्र. ५.४ ला जावे)***
5.2 Was there any cost incurred in getting tested for Covid-19 since ……….month?<br>............ महिन्यापासून कोविड-१९ चाचणी करून घेण्यासाठी काही खर्च आला का?	1. Yes 2. No १. हो २. नाही<br>***(If No, go to Q 5.3) (जर नाही, तर प्र. ५.३ ला जावे)***
5.2.1 If Yes, how much money was spent on testing?<br>जर होय, तर चाचणीसाठी किती पैसे खर्च केले गेले?	_____ (in INR) ______ (भारतीय रुपयांमध्ये)
5.3 Has any member of your household been ever tested in a Covid-19 village testing camp since ……….month? ....महिन्यापासून, तुमच्या घरातील एखाद्या सदस्याची कोविड-१९ गाव चाचणी शिबिरात कधी चाचणी झाली आहे का?	1. Yes 2. No 3. No testing camp was available<br><br>१. हो २ नाही ३. चाचणी शिबिर उपलब्ध नव्हते
5.4 How many members of the household contracted Covid-19 since ……….month?<br>.............महिन्यापासून, तुमच्या घरातील किती सदस्यांना कोविड-१९ ची लागण झाली आहे?	0 1 2 3 4 5<br>***(If 0, go to 5.14)***<br>० १ २ ३ ४ ५<br>***(० असल्यास, प्र. ५.१४ ला जावे)***
5.4.1 If any member contracted Covid-19, what support did you or your family member receive from Gram Panchayat & VTF?<br>(Query Verbatim and multi-select)	1. Food जेवण<br>2. Medicines औषधे<br>3. Counselling/ Guidance<br>समुपदेशन/ मार्गदर्शन

जर घरातील सदस्यांना कोविड-१९ ची लागण झाली असेल, तर तुम्हाला किंवा तुमच्या कुटुंबातील सदस्यांना त्यावेळी ग्रामपंचायत आणि ग्राम कृती दलांकडून/ पथकांकडून (व्हीटीएफकडून) कोणते सहकार्य मिळाले?<br>(बहुपर्यायी- मुलाखातदाराने दिलेल्या उत्तरातून विविध पर्यायांना सेलेक्ट करा)	4. Testing support for other HH members<br>इतर कुटुंबातील सदस्यांच्या चाचणीसाठी सहकार्य<br>5. Transport facility for testing or treatment<br>चाचणी किंवा उपचारांसाठी वाहतुकीची सुविधा<br>6. Facilitation of Dairy or farm produce to the market<br>डेअरी किंवा शेतमालाची बाजारात सोय<br>7. Other इतर<br>8. None काहीही नाही
5.5 Has any member of your household been isolated in a village Covid-19 isolation / care centre since ……….month?<br>.............महिन्यापासून, तुमच्या घरातील एखाद्या सदस्याला कधी गावातील कोविड-१९ विलगीकरण/ मदत केंद्रात (केअर सेंटरमध्ये) विलगीकरणात ठेवण्यात आले आहे का?	1. Yes 2. No 3. No village isolation centre was in existence<br>***(If No OR No village isolation centre, go to Q 5.6)***<br>१. हो २. नाही ३. गावात विलगीकरण केंद्र अस्तित्वात नव्हते<br>***(जर नाही किंवा गावात विलगीकरण केंद्र नाही, तर प्र. ५.६ ला जावे)***
5.5.1 If Yes, whether following facilities were available at Covid-19 isolation / care centre?<br>जर हो, तर कोविड -19 विलगीकरण/ मदत केंद्रात (केअर सेंटरमध्ये) खालील सुविधा उपलब्ध होत्या का?<br>(Not verbatim but multi-select- Check/ Ask each option)<br>(बहुपर्यायी- प्रत्येक पर्याय विचारा आणि त्यानुसार पर्यायांना सेलेक्ट करा)	1. Food जेवण<br>2. Hot Water गरम पानी<br>3. Sanitation स्नानगृह<br>4. Electricity वीज<br>5. Masks मास्क<br>6. Sanitizers सॅनिटायझर<br>7. Toilet प्रसाधनगृह<br>8. Entertainment resources (TV, Books etc.) करमणुकीची साधने (टीव्ही, पुस्तके इ.)<br>9. Other इतर<br>10. None of the above वरीलपैकी काहीही नाही
5.6 How many members of the household were hospitalized due to Covid-19 since ……….month?<br>.............महिन्यापासून, तुमच्या घरातील किती सदस्यांना कोविड-१९ मुळे रुग्णालयात भरती करावे लागले आहे?	0 1 2 3 4 5<br>***(If 0, go to 5.14)***<br>० १ २ ३ ४ ५<br>***(० असल्यास, प्र. ५.१४ ला जावे)***
5.7 Hospitalization was in which facility?<br>कोणत्या रुग्णालयात भरती करावे लागले?	1. Government Hospital at block/ district level<br>जिल्हा/ तालुका पातळीवरील सरकारी रुग्णालय (दवाखाना)<br>2. Government PHC/CHC within village<br>गावामधील सरकारी प्राथमिक आरोग्य केंद्र/ सामुदायिक आरोग्य केंद्र<br>3. Government PHC/CHC outside village<br>गावाबाहेरील सरकारी प्राथमिक आरोग्य केंद्र/ सामुदायिक आरोग्य केंद्र<br>4. CCC within village<br>गावातील कोविड मदत केंद्र<br>5. CCC outside village<br>गावाबाहेरील कोविड मदत केंद्र<br>6. Private खाजगी

	7. Aided Hospital अनुदानित रुग्णालय
5.8 Did any member of your household require oxygen support at home to recover from Covid-19 since ……….month?<br>.............महिन्यापासून, तुमच्या घरातील एखाद्या सदस्याला कोविड-१९ मधून बरे होण्याकरिता घरी ऑक्सिजनची गरज लागली का?	1. Yes हो<br>2. No नाही
5.9 How much money was spent in treatment of Covid-19 illness in your household since ……….month?<br>.............महिन्यापासून, तुमच्या घरात कोविड-१९ रोगाच्या उपचारासाठी किती पैसे खर्च झाले?	_____ (in INR)<br>______ (भारतीय रुपयांमध्ये)
5.10 How much satisfied were you with the treatment received for Covid-19?<br><br>कोविड-१९ साठी मिळालेल्या उपचाराबाबत तुम्ही किती समाधानी आहात?	1. Very satisfied अतिशय समाधानी<br>2. Satisfied समाधानी<br>3. Neutral तटस्थ<br>4. Dissatisfied असमाधानी<br>5. Very dissatisfied अतिशय असमाधानी
5.11 Has there been any death in the household due to Covid-19 since ……….month?<br>.............महिन्यापासून, तुमच्या घरात कोविड-१९ मुळे एखादा मृत्यू झाला आहे का?	1. Yes 2. No<br>If yes, how many………….<br>***(If No, go to 5.13)***<br>१. हो २. नाही<br>हो असल्यास, किती? _____<br>***(जर नाही, तर प्र. ५.१३ ला जावे)***
5.12 List of Covid-related government schemes कोविड संबंधित शासकीय योजना<br>1. Financial Support for children in the age group of 0 to 18 years who have lost both parents<br>दोन्ही पालक गमावलेल्या वर्षे वयोगटातील 1८ ते 0 बालकांसाठीअर्थसहाय्य<br>2. Ex-gratia assistance to anganwadi/staff who die due to COVID-19 while performing covid-19 related duties<br>कोविड - सबंधित कर्तव्य बजावत असताना कोविड १९-१९ मुळे मृत्यु होणाऱ्या अंगणवाडी कर्मचारी यांना सानुग्रह/सहाय्य<br>3. Mission Vatsalya Yojana<br>मिशन वात्सल्य योजना<br>4. 50,000 Rs. Financial Help if any family member dies due to Covid<br>कोविडमुळे घरातील कोणी मृत्यू पावल्यास ५०,००० रु. सानुग्रह सहाय्य	1. Aware but not applied 2. Aware and applied 3. Not aware<br>1. माहिती आहे पण अर्ज नाही केला 2. माहिती आहे आणि अर्ज केला 3. माहिती नाही<br>1. Aware but not applied 2. Aware and applied 3. Not aware<br>1. माहिती आहे पण अर्ज नाही केला 2. माहिती आहे आणि अर्ज केला 3. माहिती नाही<br>1. Aware but not applied 2. Aware and applied 3. Not aware<br>1. माहिती आहे पण अर्ज नाही केला 2. माहिती आहे आणि अर्ज केला 3. माहिती नाही<br>1. Aware but not applied 2. Aware and applied 3. Not aware<br>1. माहिती आहे पण अर्ज नाही केला 2. माहिती आहे आणि अर्ज केला 3. माहिती नाही
5.12.1 If applied to government scheme, then did you get the benefit?<br>जर कोविड संबंधित शासकीय योजनेसाठी अर्ज केला आहे तर तुम्हाला योजनेचा लाभ मिळाला आहे का?	1. Yes 2. No<br>१. हो २. नाही

5.12.1.1 If applied, what support did you receive from Gram Panchayat & VTF in registering for government schemes? (Not verbatim but multi-select)<br>जर हो, तर तुम्हाला ग्रामपंचायत आणि ग्राम कृती दलांकडून/ पथकांकडून (व्हीटीएफकडून) सरकारी योजनांचा लाभ घेण्यासाठी काय मदत मिळाली?<br>(बहुपर्यायी- प्रत्येक पर्याय विचारा आणि त्यानुसार पर्यायांना सेलेक्ट करा)	1. Provided information of the scheme<br>योजनेची माहिती दिली<br>2. Helped in filling the form<br>फॉर्म भरण्यास मदत केली<br>3. Helped in arranging documents<br>कागदपत्रांची व्यवस्था करण्यास मदत केली<br>4. Other इतर
5.13 Has any member of your household experienced Covid-19 related stigma or discrimination since ……….month?<br>.............महिन्यापासून तुमच्या कुटुंबातील एखाद्या सदस्याने कोविड-१९ संदर्भात अपमानास्पद वागणूक/ भेदभाव अनुभवला आहे का?	1. Yes हो<br>2. No नाही<br>3. Not sure निश्चित सांगू शकत नाही
5.14 Have you observed any resident of the village facing Covid-19 related stigma or discrimination since ……….month?<br>.............महिन्यापासून तुम्ही गावातील एखाद्या व्यक्तीला कोविड-१९ संदर्भात अपमानास्पद वागणूक/ भेदभाव अनुभवताना पाहिले आहे का?	1. Yes हो<br>2. No नाही<br>3. Not sure निश्चित सांगू शकत नाही

VI. SUPPORT AND CONTAINMENT- पाठिंबा व प्रतिबंध

6.1 Did the ASHA come for a household survey at your home?<br><br>आशा तुमच्या घरी घरगुती सर्वेक्षणासाठी आली होती का?	1. Frequently अनेकदा<br>2. Sometimes कधीकधी<br>3. Rarely क्वचित<br>4. Never कधीही नाही
6.2 Since ……….month, have you received soap from the government / PHC / panchayat committee/ NGO?<br>.............महिन्यापासून, तुम्हाला शासन/ प्राथमिक आरोग्य केंद्र/पंचायत समिती/ स्वयंसेवी संस्था यांच्याकडून साबण मिळाला आहे का?	1. Frequently अनेकदा<br>2. Sometimes कधीकधी<br>3. Rarely क्वचित<br>4. Never कधीही नाही
6.3 Since ……….month, have you received sanitizer from the government / PHC / panchayat committee/ NGO?<br>.............महिन्यापासून, तुम्हाला शासन/ प्राथमिक आरोग्य केंद्र/पंचायत समिती/ स्वयंसेवी संस्था यांच्याकडून सॅनिटायझर मिळाला आहे का?	1. Frequently अनेकदा<br>2. Sometimes कधीकधी<br>3. Rarely क्वचित<br>4. Never कधीही नाही
6.4 Since ……….month, have you received masks from the government / PHC / panchayat committee/ NGO?<br>.............महिन्यापासून, तुम्हाला शासन/ प्राथमिक आरोग्य केंद्र/पंचायत समिती/ स्वयंसेवी संस्था यांच्याकडून मास्क मिळाले	1. Frequently अनेकदा<br>2. Sometimes कधीकधी<br>3. Rarely क्वचित<br>4. Never कधीही नाही

आहेत का?	
6.5 Since ……….month, have you received any AYUSH immunity boosters from the government / PHC / panchayat committee/ NGO?<br>.............महिन्यापासून, तुम्हाला शासन/ प्राथमिक आरोग्य केंद्र/पंचायत समिती/ स्वयंसेवी संस्था यांच्याकडून काही आयुष प्रतिकारशक्ती वर्धक मिळाले आहेत का?	1. Frequently अनेकदा<br>2. Sometimes कधीकधी<br>3. Rarely क्वचित<br>4. Never कधीही नाही
6.6 Have you received any support from **village panchayat** / CFV Village Task Force in management of Covid-19 illness?<br>(Query verbatim and Multiple options permitted)<br><br>तुम्हाला गाव पंचायत/ कोविड-मुक्त गाव ग्राम कृती दल यांच्याकडून कोविड-१९ आजार व्यवस्थापनात मदत मिळाली आहे का?<br>(बहुपर्यायी- मुलाखातदाराने दिलेल्या उत्तरातून विविध पर्यायांना सेलेक्ट करा)	1. Testing चाचण्यांमध्ये<br>2. Vaccination लसीकरण<br>3. Hospitalization रुग्णालयात भरती करण्यामध्ये<br>4. Oxygen support for home management आजारावर घरी उपचार चालू असल्यास ऑक्सिजनची व्यवस्था<br>5. Oximeter for home isolation गृह विलगीकरणात ऑक्सिमीटरची व्यवस्था<br>6. Thermometer for home isolation गृह विलगीकरणात थर्मोमीटरची व्यवस्था<br>7. Drugs for home isolation गृह विलगीकरणात औषधांची व्यवस्था<br>8. Other इतर<br>9. None काही नाही
6.7 Was it ever communicated to you by the Panchayat/PHC/VTF to install the Aarogya Setu application?<br>तुम्हाला पंचायत/ प्राथमिक आरोग्य केंद्र/ग्राम कृती दल यांच्याकडून कधी आरोग्य सेतु ॲप स्थापित करण्यास सांगण्यात आले का?	1. Yes हो<br>2. No नाही<br>3. Not aware of app ॲपविषयी माहिती नाही
6.8 Were Covid-19 testing camps available in the village since ……….month,?<br><br>.............महिन्यापासून तुमच्या गावात कोविड-१९ चाचणी शिबिरे उपलब्ध होती का?	1. Always नेहमी<br>2. Mostly बऱ्याचदा<br>3. Occasionally कधीतरी<br>4. Rarely क्वचित<br>5. Never कधीच नाही
6.9 Were Covid-Care centres available in the village since ……….month?<br><br>.............महिन्यापासून तुमच्या गावात कोविड केअर सेंटर (सीसीसी) उपलब्ध होती का?	1. Always नेहमी<br>2. Mostly बऱ्याचदा<br>3. Occasionally कधीतरी<br>4. Rarely क्वचित<br>5. Never कधीच नाही
6.10 Did the eligible children in your locality/household receive regular under-3 immunization services in the PHC/HWC since ……….month?<br><br>.............महिन्यापासून तुमच्या परिसरातील/ घरातील पात्र लहान मुलांना ३ वर्षांच्या आतील नियमित लसीकरण सेवा मिळाली का?	1. Always नेहमी<br>2. Mostly बऱ्याचदा<br>3. Occasionally कधीतरी<br>4. Rarely क्वचित<br>5. Never कधीच नाही

6.11 Did pregnant women in your locality/household receive regular antenatal care services in the PHC/HWC since ……….month?<br><br>.............महिन्यापासून, तुमच्या परिसरातील / घरातील गर्भवती महिलांना प्राथमिक आरोग्य केंद्र/ आरोग्य व स्वास्थ्य केंद्र येथे नियमित प्रसूतिपूर्व काळजी सेवा प्राप्त करणे शक्य होते का?	1. Always नेहमी<br>2. Mostly बऱ्याचदा<br>3. Occasionally कधीतरी<br>4. Rarely क्वचित<br>5. Never कधीच नाही
6.12 Did pregnant women in your locality/household have access to ambulance / emergency transport since ……….month?<br><br>.............महिन्यापासून, तुमच्या परिसरातील / घरातील गर्भवती महिलांना अॅब्युलन्स/ आपत्कालीन वाहतूक प्राप्त करणे शक्य होते का?	1. Always नेहमी<br>2. Mostly बऱ्याचदा<br>3. Occasionally कधीतरी<br>4. Rarely क्वचित<br>5. Never कधीच नाही
6.13 Did patients with chronic diseases (DM/HTN/COPD/etc) in your locality/household have access to medications since ……….month?<br><br>.............महिन्यापासून, तुमच्या परिसरातील / घरातील दीर्घकालीन आजारी व्यक्तींना औषधे प्राप्त करणे शक्य होते का?	1. Always नेहमी<br>2. Mostly बऱ्याचदा<br>3. Occasionally कधीतरी<br>4. Rarely क्वचित<br>5. Never कधीच नाही
6.14 Whether you or any member from your household participated in any of the following activities in the village?<br>(Multi-select and check for every option)<br><br>आपण किंवा आपल्या घरातील कोणत्याही सदस्याने गावातील खालीलपैकी कोणत्याही कार्यात भाग घेतला आहे का?<br>(बहुपर्यायी- प्रत्येक पर्याय विचारा आणि त्यानुसार पर्यायांना सेलेक्ट करा)	1. Household Survey- Tracing<br>घरगुती सर्वेक्षण- संशयित रुग्ण शोधणे (ट्रेसिंग)<br>2. Covid Testing कोविड चाचणी शिबिरे (टेस्टिंग)<br>3. Covid Vaccination कोविड लसीकरण<br>4. Awareness generation जनजागृती मोहीम<br>5. Facilitation of govt. schemes etc.<br>सरकारी योजनांची लाभ मिळवून देण्यामध्ये<br>6. Provided funds for formation of Corona Village Committee or formation of Covid care centre or awareness generation<br>कोरोना ग्राम समिती स्थापन करण्यासाठी किंवा कोविड केअर/ विलगीकरण कक्ष स्थापन करण्यासाठी किंवा जनजागृतीसाठी निधी उपलब्ध करून देण्यामध्ये<br>7. Arrange transport facility for testing, vaccination, treatment etc.<br>चाचणी (टेस्टिंग), लसीकरण, उपचार इ. साठी वाहतुकीची व्यवस्था<br>8. Ration/ Medicine/ Kit distribution<br>राशन/ औषध/ किट वाटप<br>9. Other इतर<br>10. None कोणत्याही नाही
