# Supplementary material for: COVID-19 Resilience and Risk Reduction Intervention in Rural Populations of Western India: Retrospective Evaluation
Source: JMIR Public Health Surveill. 2024 Jul 29;10:e47520. doi: 10.2196/47520 (PMC11319881; doi:10.2196/47520)
Supplement: Multimedia Appendix 4 [file publichealth_v10i1e47520_app4.docx]

**Supplementary Tables**

**Table S1- Selected villages and households in Pune and Satara**

| **Group number** | **Total No of villages to be covered** | **No of Household to be covered per village in Pune** | **No of Household to be covered per village in Satara** |
| --- | --- | --- | --- |
| Group 1 | 7 | 54 | 71 |
| Group 2 | 4 | 94 | 125 |
| Group 3 | 3 | 125 | 167 |
| Group 4 | 2 | 188 | 251 |
|  | Total Village=16 | Total Household = 1505 | Total Household = 2000 |

**Table S2- Balancing property**

| **Variable Name** |  | **Mean** | | **%bias** | **%reduct bias** | **t-test** | |
| --- | --- | --- | --- | --- | --- | --- | --- |
|  |  | **Intervention** | **Control** |  |  | **t** | **p>t** |
| Number of family members | Unmatched | 4.894 | 4.412 | 20.2 |  | 6.14 | 0 |
|  | Matched | 4.800 | 4.830 | -1.2 | 93.8 | -0.33 | 0.7 |
|  |  |  |  |  |  |  |  |
| Square of number of family members | Unmatched | 30.316 | 24.442 | 11.3 |  | 3.45 | 0.00 |
|  | Matched | 28.76 | 29.429 | -1.3 | 88.6 | -0.42 | 0.6 |
|  |  |  |  |  |  |  |  |
| General Category | Unmatched | 0.652 | 0.514 | 28.3 |  | 8.5 | 0 |
|  | Matched | 0.657 | 0.652 | 0.7 | 97.4 | 0.21 | 0.8 |
|  |  |  |  |  |  |  |  |
| OBC | Unmatched | 0.155 | 0.234 | -20.3 |  | -6.06 | 0 |
|  | Matched | 0.162 | 0.169 | -1.9 | 90.7 | 0.54 | 0.5 |
|  |  |  |  |  |  |  |  |
| SC & ST | Unmatched | 0.183 | 0.178 | 1.3 |  | 0.4 | 0.6 |
|  | Matched | 0.171 | 0.168 | 1.0 | 23.6 | 0.28 | 0.7 |
|  |  |  |  |  |  |  |  |
| Nuclear family | Unmatched | 0.518 | 0.821 | -68.1 |  | -20.83 | 0 |
|  | Matched | 0.563 | 0.542 | 4.6 | 93.2 | 1.13 | 0.2 |
|  |  |  |  |  |  |  |  |
| Hindu | Unmatched | 0.935 | 0.84 | 30.3 |  | 8.94 | 0 |
|  | Matched | 0.927 | 0.927 | 0.3 | 98.9 | 0.11 | 0.9 |
|  |  |  |  |  |  |  |  |
| Christian, Jain, Sikh & Muslim | Unmatched | 0.009 | 0.03 | -15 |  | -4.4 | 0 |
|  | Matched | .0101 | 0.011 | -0.5 | 95.5 | -0.19 | 0.8 |
|  |  |  |  |  |  |  |  |
| Education graduation or above | Unmatched | 0.361 | 0.387 | -5.4 |  | -1.63 | 0.104 |
|  | Matched | 0.367 | 0,369 | -0.5 | 89.9 | -0.15 | 0.8 |
|  |  |  |  |  |  |  |  |
| Education: High School & intermediate | Unmatched | 0.429 | 0.394 | 7.2 |  | 2.16 | 0.0 |
|  | Matched | 0.421 | 0.413 | 1.5 | 79.2 | 0.41 | 0.6 |
|  |  |  |  |  |  |  |  |
| Education: Primary or middle school | Unmatched | 0.188 | 0.176 | 3.1 |  | 0.93 | 0.355 |
|  | Matched | 0.188 | 0.182 | 1.7 | 45.3 | 0.45 | 0.6 |
|  |  |  |  |  |  |  |  |
| Household with member over 60years of age | Unmatched | 0.52 | 0.587 | -13.5 |  | -4.07 | 0 |
|  | Matched | 0.532 | 0.486 | 9.4 | 30.0 | 2.55 | 0 |
|  |  |  |  |  |  |  |  |
| Female respondent | Unmatched | 0.579 | 0.425 | 31.2 |  | 9.41 | 0 |
|  | Matched | 0.544 | 0.542 | 0.5 | 98.4 | 0.13 | 0.8 |
|  |  |  |  |  |  |  |  |
| Age of respondent | Unmatched | 42.559 | 46.795 | -25.3 |  | -7.76 | 0 |
|  | Matched | 42.717 | 44.416 | -10.3 | 59.9 | -3.46 | 0 |
|  |  |  |  |  |  |  |  |
| Occupation of respondent : agriculture | Unmatched | 0.037 | 0.214 | -55.6 |  | -16.18 | 0 |
|  | Matched | 0.04 | 0.049 | -2.6 | 95.3 | -1.10 | 0.2 |
|  |  |  |  |  |  |  |  |
| Room per member of house hold | Unmatched | 0.74 | 0.779 | -7.7 |  | -2.32 | 0.021 |
|  | Matched | 0.752 | 0.747 | 0.9 | 88.8 | 0.24 | 0.8 |
|  |  |  |  |  |  |  |  |
| Household with member of age 6-18 | Unmatched | 0.494 | 0.461 | 6.5 |  | 1.96 | 0.05 |
|  | Matched | 0.489 | 0.509 | -4.0 | 38.6 | -1.09 | 0.2 |
|  |  |  |  |  |  |  |  |
| Household with a child/children under 5year | Unmatched | 0.225 | 0.187 | 9.4 |  | 2.84 | 0.00 |
|  | Matched | 0.215 | 0.211 | 0.9 | 90.3 | 0.24 | 0.8 |
|  |  |  |  |  |  |  |  |
| Household with pregnant women | Unmatched | 0.02 | 0.008 | 10.1 |  | 3.12 | 0.002 |
|  | Matched | 0.02 | 0.018 | -0.4 | 96.2 | -0.09 | 0.9 |
|  |  |  |  |  |  |  |  |
| Household with 1 or more comorbidity | Unmatched | 0.065 | 0.105 | -14.3 |  | -4.26 | 0 |
|  | Matched | 0.067 | 0.050 | 6.0 | 58.1 | 1.94 | 0 |
|  |  |  |  |  |  |  |  |
| Household has a smartphone | Unmatched | 0.938 | 0.105 | -15.4 |  | 4.58 | 0 |
|  | Matched | 0.932 | 0.911 | 7.7 | 49.8 | 2.16 | 0.0 |

| **Sample** | **Ps R2** | **LR chi2** | **p>chi2** | **Mean Bias** | **Median Bias** | **B** | **R** | **%Var** |
| --- | --- | --- | --- | --- | --- | --- | --- | --- |
| Unmatched | 0.269 | 1357.86 | 0 | 19 | 14.3 | 136.2 | 0.82 | 75 |
| Matched | 0.010 | 41.59 | 0.005 | 2.8 | 1.3 | 23.8 | 0.90 | 50 |
|  |  |  |  |  |  |  |  |  |

**Table S3-Logistic results: Dependent variable is households in Pune district (Comparison group household in Satara district)**

| **Variables** | **Odds Ratio (95% CI)** |
| --- | --- |
| Number of family members | 0.828 (0.752, 0.911) |
| Square of number of family members | 1.005 (1.001, 1.008) |
| General Category | 17.213 (9.628, 30.773) |
| OBC, | 8.920 (4.912, 16.199) |
| SC & ST | 35.546 (18.834, 67.089) |
| Nuclear family | 0.116 (0.090, 0.150) |
| Hindu | 7.155 (4.858, 10.538) |
| Christian, Jain, Sikh & Muslim | 2.889 (1.342, 6.218) |
| Education graduation or above | 1.344 (0.810, 2.230) |
| Education: High School & intermediate | 1.566 (0.951, 2.580) |
| Education: Primary or middle school | 2.009 (1.218,3.316) |
| Household with member over 60years of age | 0.553 (0.463, 0.660) |
| Household with member of age 6-18year | 0.744 (0.616, 0.898) |
| Household with a child/children under 5year | 0.909 (0.727, 1.137) |
| Household with pregnant women | 1.803 (0.878, 3.704) |
| Household has a smartphone | 0.637 (0.469, 0.865) |
| Household with 1 or more comorbidity | 0.723 (0.536, 0.975) |
| Female respondent | 3.326 (2.792, 3.961) |
| Age of the respondent | 0.984 (0.977, 0.990) |
| Occupation of respondent : agriculture | 0.056 (0.040, 0.078) |
| Room per member of house hold | 1.041 (0.859, 1.262) |
| Pseudo R-sq | 0.2705 |
| N | 3680 |

**Table S4- Summary Statistics of variables used in regression**

| **Independent Variables** | **Mean (95% CI)** |
| --- | --- |
| OBC | 0.201(0.187-0.215) |
| SC & ST | 0.173(0.160-0.186) |
| Hindu | 0.891(0.880-0.902) |
| Muslim, Sikh, Christian & Jain | 0.017(0.013-0.022) |
| No educated member in household | 0.033(0.027-0.039) |
| Highest education in the family primary and middle school | 0.186(0.172-0.199) |
| Household has a member over the age of 60 years | 0.554(0.536-0.571) |
| Respondent was female | 0.461(0.444-0.478) |
| Age of the respondent | 44.947(44.467-45.427) |
| Respondent was housewife | 0.092(0.082-0.102) |
| No. of rooms in the house per member | 0.774(0.755-0.793) |
| Household has a member of age 6 to 18years | 0.470(0.452-0.487) |
| Household has a child under 5years of age | 0.197(0.184-0.212) |
| Household has a pregnant woman | 0.013(0.009-0.016) |
| Household has a member suffering from a co-morbidity^#^ | 0.078(0.069-0.088) |
| Household has a smartphone | 0.914(0.904-0.923) |
| Any member of the household is health care worker | 0.022(0.017-0.027) |
| There is toilet in the house | 0.940(0.017-0.027) |
| Any member of household consumes tobacco | 0.111(0.101-0.122) |
| Any member of household consumes Alcohol | 0.177(0.164-0.190) |
| ASHA visited for the household survey | 0.922(0.913-0.931) |

**Table S5: Sociodemographic characteristics of study households**

|  | **Pune** | **Satara** |
| --- | --- | --- |
|  | **(N = 1637)** | **(N = 2,043)** |
| **Age of the respondent** |  |  |
| Mean±SD | 42.6± 19.11 | 46.8±13.97 |
| 18- 30 years | 398(24.31%) | 302(14.78%) |
| 31-45 years | 616(37.63%) | 715(35.00%) |
| 46-60years | 454(27.73%) | 618(30.25%) |
| 61 years and above | 169(10.32%) | 408(19.97%) |
| **Sex of respondent** |  |  |
| Female | 948 (57.9%) | 868 (42.5%) |
| Male | 689 (42.1%) | 1175 (57.5%) |
| **Religion** |  |  |
| Buddhist | 92 (5.6%) | 266 (13.0%) |
| Christian | 0 (0.0%) | 2 (0.1%) |
| Hindu | 1530 (93.5%) | 1716 (84.0%) |
| Jain | 1 (0.1%) | 0 (0.0%) |
| Muslim | 13 (0.8%) | 59 (2.9%) |
| Sikh | 1 (0.1%) | 0 (0.0%) |
| **Caste** |  |  |
| General | 1068 (65.2%) | 1051 (51.4%) |
| OBC | 253 (15.5%) | 479 (23.4%) |
| SC | 132 (8.1%) | 359 (17.6%) |
| ST | 168 (10.3%) | 5 (0.2%) |
| Other/Prefer not to say | 16 (1.0%) | 149 (7.3%) |
| **Education status** |  |  |
| Illiterate | 234 (14.3%) | 339 (16.6%) |
| Primary school certificate | 308 (18.8%) | 417 (20.4%) |
| Middle school certificate | 438 (26.8%) | 464 (22.7%) |
| High school (Matric) certificate | 378 (23.1%) | 474 (23.2%) |
| Intermediate or diploma | 378 (23.1%) | 474 (23.2%) |
| Graduate | 138 (8.4%) | 178 (8.7%) |
| Post-Graduate | 27 (1.6%) | 21 (1.0%) |
| Professional | 1 (0.1%) | 2 (0.1%) |
| **Educational status – member with the highest educational qualification** | | |
| Primary school certificate | 31 (1.9%) | 36 (1.8%) |
| Middle school certificate | 130 (7.9%) | 105 (5.1%) |
| High school (Matric) certificate | 311 (19.0%) | 310 (15.2%) |
| Intermediate or diploma | 189 (11.5%) | 285 (14.0%) |
| Graduate | 348 (21.3%) | 519 (25.4%) |
| Post Graduate | 111 (6.8%) | 117 (5.7%) |
| Professional | 9 (0.5%) | 14 (0.7%) |
| NA | 508 (31.0%) | 650 (31.8%) |
| **Employment** |  |  |
| Professional | 56 (3.4%) | 46 (2.3%) |
| Government Service | 51 (3.1%) | 42 (2.1%) |
| Private Job | 209 (12.8%) | 120 (5.9%) |
| Business / Shop owner | 58 (3.5%) | 43 (2.1%) |
| Agriculture | 1036 (63.3%) | 947 (46.4%) |
| Skilled agricultural or craft worker | 29 (1.8%) | 104 (5.1%) |
| Unskilled agricultural or craft worker | 103 (6.3%) | 203 (9.9%) |
| House Wife | 60 (3.7%) | 437 (21.4%) |
| Unemployed | 16 (1.0%) | 78 (3.8%) |
| Other | 19 (1.2%) | 23 (1.1%) |
| **Household member a healthcare worker** |  |  |
| Yes | 32 (2.0%) | 39 (1.9%) |
| **Household type** |  |  |
| Joint | 789 (48.2%) | 365 (17.9%) |
| Nuclear | 848 (51.8%) | 1678 (82.1%) |
| **Total number of rooms in the household** |  |  |
| Mean±SD | 3.06±1.93 | 2.95±1.63 |
| **Household composition** |  |  |
| Elderly | 852 (52.0%) | 1200 (58.7%) |
| Under-5 children | 369 (22.5%) | 383 (18.7%) |
| Children- 6-18years | 808 (49.4%) | 942 (46.1%) |
| Pregnant women | 32 (2.0%) | 16 (0.8%) |
| **Functional Toilet** |  |  |
| Present | 1538 (94.0%) | 1923 (94.1%) |
| **Comorbidities (present)** |  |  |
| Diabetes | 185 (11.3%) | 312 (15.3%) |
| Hypertension | 228 (13.9%) | 462 (22.6%) |

**Table S6 Effect of CFVP on awareness related variables**

| **Indicators** | **OR(CI)** | **N, Pseudo R^2^** |
| --- | --- | --- |
| **Awareness regarding COVID-19 control Program** |  |  |
| Are you aware of Village level committee or Taskforces formed at the village | 0.167(0.135-0.206) *P<.001* | N=3183 R2=0.168 |
| Awareness camps were setup in the village | 0.881(0.658-1.18) *P=.40* | N=3183 R2=0.092 |
| Subject/ focus of awareness campaigns was Nutrition & Medicines | 1.259 (1.072-1.478) *P=.005* | N=3183 R2=0.025 |
| Subject/ focus of awareness campaigns was COVID Appropriate Behaviour | 1.587(1.349-1.866) *P<.001* | N=3183 R2=0.056 |
| Subject/ focus of awareness campaigns was COVID Vaccination | 1.196(0.932-1.535) *P=.16* | N=3183 R2=0.081 |
| Subject/ focus of awareness campaigns was Govt Scheme | 2.587(1.891-3.538) *P<.001* | N=3183 R2=0.052 |
| Awareness campaigns promoted Covid Testing | 0.769(0.646-0.915) *P=.003* | N=3183 R2=0.050 |
| Posters were used to spread COVID related awareness | 1.383(1.177-1.624) *P<.001* | N=3183 R2=0.047 |
| Public Announcement were used to spread COVID related awareness | 4.979(4.186-5.922) *P<.001* | N=3183 R2=0.138 |
| Home Visit were done to spread COVID related awareness | 1.451(1.201-1.753) *P<.001* | N=3183 R2=0.053 |
| Social media were used to spread COVID related awareness | 1.723(1.46-2.033) *P<.001* | N=3183 R2=0.079 |
| Influential people were used to spread COVID related awareness | 1(0.82-1.219) *P=*0.99 | N=3183 R2=0.041 |
|  |  |  |
| **COVID-19 related awareness** |  |  |
| Awareness regarding handwash as a mean of preventing the COVID-19 infection | 5.507(3.866-7.845) *P<.001* | N=3183 R2=0.144 |
| Awareness regarding immunity boosting drugs as a mean of preventing the COVID-19 infection | 1.576(1.339-1.854) *P<.001* | N=3183 R2=0.060 |
| Awareness regarding COVID vaccination as a mean of preventing the COVID-19 infection | 4.41 (3.679-5.285) *P<.001* | N=3183 R2=0.126 |
| Awareness regarding wearing masks as a mean of preventing the COVID-19 infection | 1.249(0.717-2.177) *P=.43* | N=3061 R2=0.084 |
| Awareness regarding social distancing as a means of preventing the COVID-19 infection | 1.151(0.893-1.485) *P=.28* | N=3183 R2=0.034 |
| Awareness regarding variant | 0.126 (0.103-0.154  ) *P<.001* | N=3183 R2= 0.235 |
| COVID-19 is a serious illness | 1.306 (1.067-1.598) *P=.01* | N=3183 R2=0.025 |
|  |  |  |
| **Arogya Setu & its usage** |  |  |
| Had Arogya Setu application installed in their smart phone | 0.888(0.732-1.078) *P=.23* | N=2908,  R ^2^=0.072 |
| Used Arogya Setu app for Contact tracing | 0.683(0.528-0.885) *P=.004* | N=2908,  R ^2^=0.095 |
| Used Arogya Setu app for self-assessment and understanding the risk of infection status. | 1.22 (0.989-1.515) *P=.06* | N=2859,  R ^2^=0.0592 |
| Used Arogya Setu app for getting the lists of testing facilities and COVID test results. | 1.090 (0.857- 1.386) *P=.48* | N=2859,  R ^2^=0.0604 |
| Used Arogya Setu app for knowing updates, advisory & best practices related to COVID-19. | 1.532 (1.231- 1.908) *P<.001* | N=2859,  R ^2^=0.0720 |
|  |  |  |
| **Testing** |  |  |
| Has any member of your household been ever tested (Antigen/RTPCR) for Covid-19 | 1.322(1.126-1.553)  *P=.001* | N=3183,  R ^2^=0.0385 |
| Covid-19 testing camps available in the village | 0.160 (0.133-0.191) *P<.001* | 3183, 0.1792 |
| Observed Covid-19 related stigma or discrimination | 0.317 (0. 250 -0.402) *P<.001* | N=3183 R2= 0.183 |
|  |  |  |
| **Other medical services** |  |  |
| Eligible children in your locality/household receive regular under-3 immunization | 1.181** (1.515-2.184) | N=3183,  R ^2^=0.0463 |
| pregnant women in your locality/household receive regular antenatal care service | 1.980 (1.674-2.342) *P<.001* | N=3183,  R ^2^=0.0420 |
| Pregnant women in the locality/household have access to ambulance / emergency transport | 1.538(1.309-1.806) *P<.001* | N=3183,  R ^2^=0.037 |
| Patients with chronic disease has access to medicines | 2.498(2.122-2.942) *P<.001* | N=3183,  R ^2^=0.0499 |

**Table S7-Sub-group analysis - awareness indices**

| **Index for program awareness** | | **Index for COVID-19 awareness** | | **Index for Combined awareness** | |
| --- | --- | --- | --- | --- | --- |
| Caste SC & ST | Education up to Middle School | Caste SC & ST | Education up to Middle School | Caste SC & ST | Education up to Middle School |
| 0.002  *P=.99* | 0.691 *P<.001* | 0.454  *P<.001* | 0.882 *P<.001* | 0.225  *P=.21* | 0.959 *P<.001* |
| 551 | 696 | 551 | 696 | 551 | 686 |
| 0.228 | 0.194 | 0.227 | 0.251 | 0.216 | 0.234 |

Notes: = Figures in parentheses are 95% confidence interval

**Table S8-Sub-group analysis – Other Indicators**

| **Indicators** | **Caste: ST & SC** | | **Education level up to middle school** | |
| --- | --- | --- | --- | --- |
|  | **OR(CI)** | **N, Pseudo R^2^** | **OR(CI)** | **N, Pseudo R^2^** |
| **Perceived effect of COVID-19 on comorbid** | | | | |
| Covid-19 disease is more serious in people with Heart disease | 1.444 (0.932-2.236)  *P=.10* | N=551 R2=0.042 | 1.095(0.736-1.629) *P=.64* | N=695 R2=0.033 |
| Covid-19 disease is more serious in people with Diabetes | 1.154(0.756-1.762)  *P=.51* | N=551 R2=0.060 | 1.2(0.808-1.782) *P=.37* | N=695 R2=0.086) |
| Covid-19 disease is more serious in people with Hypertension | 2.960( 1.953- 4.484) *P<.001* | N=551 R2= 0.084 | 5.915(3.726- 9.390) *P<.001* | N=695 R2= 0.164 |
| Covid-19 disease is more serious in people with lung disease | 1.86(0.756-1.762)  *P=.005* | N=551 R2=0.097 | 2.706(1.709-4.283) *P<.001* | N=695 R2=0.081 |
| Covid-19 disease is more serious in people with low immunity | 1.08(0.713-1.636)  *P=.72* | N=551 R2=0.091 | 2.586(1.713-3.902) *P<.001* | N=695 R2=0.117 |
| **Use of preventive measures** | |  |  |  |
| Wash your hands with soap/ sanitizer at least 4 times a day | 1.544(1.008-2.366)  *P=.046* | N=546 R2=0.078 | 1.225(0.831-1.806)/  *P=.30* | N=695 R2=0.086 |
| Wear the mask while leaving the house | 1.95(1.297-2.932)  *P=.001* | N=551 R2=0.060 | 1.409(0.97-2.045) *P=.07* | N=695 R2=0.052 |
| Faced any difficulty in accessing or affording Soap | 1.125(0.453-2.792)  *P=.80* | N=542 R2=0.170 | 0.339(0.184-0.625) *P=.001* | N=681 R2=).098 |
| Faced any difficulty in accessing or affording Sanitizer | 0.507(0.245-1.052)  *P=.07* | N=542 R2=0.124 | 0.408(0.246-0.676) *P<.001* | N=691 R2=0.060 |
| Faced any difficulty in accessing or affording Mask | 0.54(0.256-1.136)  *P=.10* | N=542 R2=0.121 | 0.352(0.202-0.614) *P<.001* | N=691 R2=0.071 |
|  |  |  |  |  |
| **Arogya Setu & its usage** | |  |  |  |
| Had Arogya Setu application installed in their smart phone | 1.084(0.653-1.797)  *P=.76* | N=483 R2=0.146 | 0.927(0.446-1.926) *P<.84* | N=539 R2=0.158 |
| Used Arogya Setu app for Contact tracing | 0.85(0.468-1.543)  *P=.60* | N=479 R2=0.160 | 0.425(0.174-1.037) *P=.06* | N=539 R2=0.204 |
| Used Arogya Setu app for self-assessment and understanding the risk of infection status. | 1.02(0.590-1.764)  *P=.94* | N=483 R2=0.129 | 2.331(0.904-6.014) *P<.08* | N=481 R2=0.136 |
| Used Arogya Setu app for getting the lists of testing facilities and COVID test results. | 0.942(0.49-1.812)  *P=.86* | N=483 R2=0.193 | 1.538(0.506-4.677) *P=.49* | N=445 R2=0.133 |
| Used Arogya Setu app for knowing updates, advisory & best practices related to COVID-19. | 1.474(0.844-2.572)  *P=.17* | N=483 R2=0.125 | 2.865(1.02-8.042) *P=.046* | N=481 R2=0.146 |
|  |  |  |  |  |
| **Testing** |  |  |  |  |
| Has any member of your household been ever tested (Antigen/RTPCR) for Covid-19 | 1.828(1.218-2.744)  *P=.004* | N=511 R2=0.051 | 2.09(1.421-3.074) *P<.001* | N=695 R2=0.056 |
| Covid-19 testing camps available in the village | 0.157(0.097-0.253)  *P<.001* | N=551 R2=0.219 | 0.129(0.079-0.211) *P* <.001 | N=695 R2=0.248 |
|  |  |  |  |  |
| **Use of Vaccination Facilities** |  |  |  |  |
| The first dose of the COVID-19 vaccine for the eligible household members of the age 60years and above was administered at the government facility | 0.074(0.045-0.122)  *P<.001* | N=551 R2=0.235 | 0.294(0,199-0,432) *P<.001* | N=691 R2=0.102 |
| The first dose of the COVID-19 vaccine for the eligible household members of the age 60years & above was administered at the village vaccination camp | 1.355(0.839-2.189)  *P=.21* | N=511 R2=0.120 | 0.793(0.518-1.212) *P=.28* | N=695 R2=0.112 |
| The first dose of the COVID-19 vaccine for the eligible household members below the age of 60years was administered at the government facility | 0.166(0.106-0.216)  *P<.001* | N=551 R2=0.142 | 0.407(0.277-0.597) *P<.001* | N=695 R2=0.068 |
| The first dose of the COVID-19 vaccine for the eligible household members below the age of 60years was administered at the village vaccination camp | 10.021(6.2-16.195)  *P<.001* | N=551 R2=0.192 | 2.378(1.627-3.476)  *P<.001* | N=695 R2=0.70 |
| The transportation was provided for the villagers if the site of vaccination was over 1km | 3.683( 1.028- 13.206)  *P=.045* | N=407 R2=0.119 | 1.561(0.752-3.241) *P=.045* | N=681 R2=0.091 |
| **Other medical services** |  |  |  |  |
| Eligible children in your locality/household receive regular under-3 immunization | 0.881(0.543-1.431)  *P=.61* | N=547 R2=0.074 | 1.964(1.29-2.988)  *P=.002* | N=691 R2=0.096 |
| pregnant women in your locality/household receive regular antenatal care service | 2.938(1.887-4.576)  *P<.001* | N=547 R2=0.090 | 1.821(1.24-2.673)  *P=.002* | N=695 R2=0.067 |
| Pregnant women in the locality/household have access to ambulance / emergency transport | 1.337(0.876-2.043)  *P=.18* | N=547 R2=0.095 | 1.1642(1.119-2.41)  *P=.01* | N=695 R2=0.078 |
| Patients with chronic disease has access to medicines | 2.886(1.901-4.383)  *P<.001* | N=546 R2=0.087 | 2.654(1.791-3.934)  *P< P.001* | N=695 R2=0.093 |

Notes: Figures in parentheses are 95% confidence interval
